# Structural measures of similarity and complementarity in complex networks

**DOI:** 10.1038/s41598-022-20710-w

**Published:** 2022-10-04

**Authors:** Szymon Talaga, Andrzej Nowak

**Affiliations:** 1 Robert Zajonc Institute for Social Studies, University of Warsaw, Stawki 5/7, 00-183 Warsaw, Poland; 2 Faculty of Psychology, University of Warsaw, Stawki 5/7, 00-183 Warsaw, Poland; 3 Department of Psychology, Florida Atlantic University, 777 Glades Rd, Boca Raton, FL 33431 USA

**Keywords:** Computational science, Biochemical networks, Complexity, Human behaviour

## Abstract

The principle of similarity, or homophily, is often used to explain patterns observed in complex networks such as transitivity and the abundance of triangles (3-cycles). However, many phenomena from division of labor to protein-protein interactions (PPI) are driven by complementarity (differences and synergy). Here we show that the principle of complementarity is linked to the abundance of quadrangles (4-cycles) and dense bipartite-like subgraphs. We link both principles to their characteristic motifs and introduce two families of coefficients of: (1) structural similarity, which generalize local clustering and closure coefficients and capture the full spectrum of similarity-driven structures; (2) structural complementarity, defined analogously but based on quadrangles instead of triangles. Using multiple social and biological networks, we demonstrate that the coefficients capture structural properties related to meaningful domain-specific phenomena. We show that they allow distinguishing between different kinds of social relations as well as measuring an increasing structural diversity of PPI networks across the tree of life. Our results indicate that some types of relations are better explained by complementarity than homophily, and may be useful for improving existing link prediction methods. We also introduce a Python package implementing efficient algorithms for calculating the proposed coefficients.

## Introduction

The structure of complex networks commonly reflects their functional properties as well as mechanisms or processes that created them. Seminal studies have shown that different systems, from neural networks to the World Wide Web, tend to be characterized by the presence of statistically over-represented small subgraphs, known as network motifs^1–3^. While one may expect different motifs to be related to particular functions or properties of a given system, it is often not easy to determine what they are exactly. In some cases and specific contexts, such as gene regulatory networks, the roles played by different motifs may be revealed through experimental studies^2,4^. However, general principles that would explain the prevalence of specific motifs across different application domains are still mostly unknown.

**Figure 1:**
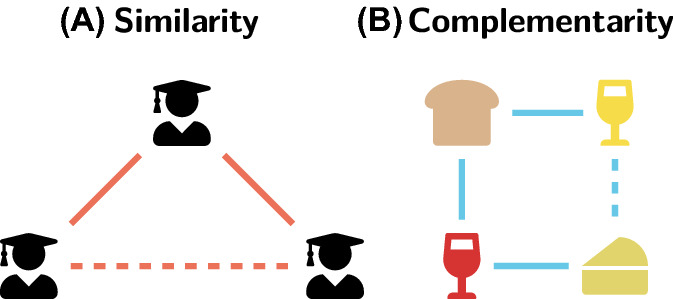
Intuitive meaning of similarity and complementarity. (**A**) If three persons are similar (e.g. they are all scientists) and we know that one of them (top) knows the other two (bottom) it is quite likely that they know each other too (dashed line). Thus, the relation is **transitive**. (**B**) If one wine (red) goes well with (is complementary to) bread and cheese and another wine (white) also goes well with the bread, then it is likely that it is a good match for the cheese too (dashed line). However, this does not imply that both wines will be drunk together, so the relation is **not transitive**.

An important exception is the widely-known abundance of triangles (3-cycles) in many types of real-world networks, which has been shown to be a structural signature of transitive relations driven by similarity between nodes in some (possibly latent) metric space^5–7^. The importance of similarity and its impact on the structure of social networks has been recognized in sociology for a long time, as it is linked to homophily and triadic closure^8–12^. While it is usually hard to disentangle their effects^13,14^, these two processes are also inherently linked as they lead to high structural equivalence^15^ between connected nodes. In other words, in similarity-driven systems two adjacent nodes are likely to share a lot of neighbors (Fig. 1A), and this implies the abundance of triangles and a latent geometric structure^7,16^.

An alike, even if less known, phenomenon is the connection between the abundance of quadrangles (4-cycles) and networks with so-called functional structure^17^, in which two nodes interact not because they are similar, but rather because one of them is similar (in some salient way) to the neighbors of the other^18^. This linkage principle leads to markedly different local connectivity structures than those found in networks dominated by triangles (e.g. typical social networks) and is characteristic for relations driven by complementarity, or differences and synergies, between the features of connected elements^17–19^.

This observation is important as many phenomena across different application domains, from cooperation, business interactions and division of labor^17,20–24^, to the quality of romantic relationships^25^, consumer choices^26^ and at least some types of protein–protein binding^18^, may indeed be better explained by the principle of complementarity than similarity. For instance, two types of wine may be often bought together with the same kinds of bread and cheese, but rarely both of them will occur in the same transaction. In other words, in this situation a wine is complementary to the bread and cheese, but not to the other wine (Fig. 1B). More generally, complementarity can be seen as a particular interpretation of the principle of heterophily, which is a preference for connecting to others who are different with respect to some salient attributes^22^.

Here we show that the principle of complementarity, unlike the more general notion of heterophily, has a straightforward geometric interpretation which links it to quadrangles as its characteristic motif, in the same way as the intrinsic geometry of similarity links it to triangles. We also show that under a particular quadrangle definition (4-cycle without diagonal shortcuts) the principle of complementarity is connected to locally dense subgraphs of high bipartivity^27^, which, again, is analogous to how the abundance of triangles implies the presence of dense unipartite subgraphs. More generally, we argue that both similarity and complementarity are important relational principles shaping the structure of networks across different application domains and provide a generic explanation for some of the prevalent structural patterns observed in many real-world systems.

In order to formalize our analysis, we first define a general family of similarity coefficients measuring the abundance of triangles at the levels of individual nodes and edges as well as entire graphs. The coefficients generalize the notions of local clustering and closure^28,29^ and therefore capture the full spectrum of transitive, similarity-driven structures. Then, starting from a simple geometric model of complementarity, we follow the same logic as in the case of similarity and define an analogous family of complementarity coefficients measuring the abundance of quadrangles.

We will call the proposed measures *structural coefficients* because they will not be defined with respect to node attributes, latent or observed, but to how different nodes are embedded in the network. Moreover, they will not measure (dis)similarity between nodes, as this problem is usually addressed by measures of *structural equivalence*^15^. Instead, structural coefficients will measure the extent to which any given edge, node or graph is compatible with the principle of similarity or complementarity. However, to facilitate the interpretation we will also show how the proposed notions of structural similarity and complementarity are related to structural equivalence.

We study the behavior of structural coefficients in some of the most important random graph models as well as multiple real-world social and biological networks. We demonstrate that they are related to meaningful domain-specific phenomena and can be used to distinguish between different types of networks. In particular, using a collection of comparable real-world networks measuring friendship and health advice ties, we show that structural coefficients discriminate effectively between social relations driven by similarity and complementarity, which provides evidence for the theoretical validity of our approach. We also demonstrate how the coefficients may be used to measure the increasing structural diversity of protein-protein interactions (PPI) across the tree of life based on hundreds of interactome networks of different organisms.

Our work complements the rich literature on network motifs, network geometry and local connectivity structures as well as introduces principled theory and methods linking different types of relations to their observable structural signatures. We argue that the customary assumption of homophily is not adequate for some types of social relations, which are better explained by complementarity, and provide tools for identifying such systems, bringing more nuance to the field of social network analysis. Moreover, the framework we propose could be, in principle, used for improving existing link prediction methods by helping to determine when the assumption of 2-path (L2/triadic) or 3-path (L3/tetradic) closure^18^ is more appropriate. Last but not least, all methods introduced in this paper are implemented in a Python package called pathcensus (see “Materials and methods” section).

### Notation and technical remarks

In this paper we consider simple undirected and unweighted graphs $G = (V, E)$ with no self-loops. We use $n = |V|$ and $m = |E|$ to denote the numbers of nodes and edges in *G* respectively. Elements of the adjacency matrix of *G* will be denoted by $a_{ij}$ and assumed to be equal to 1 if the edge (*i*, *j*) exists and 0 otherwise. For any node $i \in V$ we denote its degree by $d_i$ and its *k*-hop neighborhood by ${\mathscr {N}}_k(i)$, in particular 1-hop neighborhood will be denoted by ${\mathscr {N}}_1(i)$ (a *k*-hop neighborhood consists of nodes connected to *i* by a shortest path of length *k*) . Moreover, we will use $n_{ij} = |{\mathscr {N}}_1(i) \cap {\mathscr {N}}_1(j)|$ to denote the number of shared neighbors between nodes *i* and *j*. Averaged quantities will be denoted by diamond brackets. For instance, $\langle d_i\rangle$ will denote average node degree.

### Structural equivalence

We briefly introduce the notion of *structural equivalence*, to which we will refer at multiple points throughout the paper. Structural equivalence is a measure of the extent to which two nodes are similarly embedded in a network. It can be defined in multiple ways, but all definitions try to quantify similarity between 1-hop neighborhoods of two nodes^15^. Here we will follow a common approach and define structural equivalence in terms of Sørenson Index or normalized Hamming similarity:

1 $$\begin{aligned} H_{ij} = \frac{2n_{ij}}{d_i + d_j} \end{aligned}$$

which is also often used as an index for predicting missing links (under the assumption of triadic closure)^30^. Crucially, the notion of structural equivalence applies to pairs of nodes (not necessarily connected) and is concerned with the degree of (dis)similarity of their 1-hop neighborhoods. This is in contrast to structural coefficients we propose, which are descriptors of edges, nodes or graphs capturing the degree to which they are compatible with the logic of similarity or complementarity.

## Theory and definitions

Here we present the proposed theory of structural similarity and complementarity and introduce all the main definitions that will be used throughout the paper. We first discuss structural similarity and its nodewise and global coefficients and then define the analogous complementarity coefficients. In the second part of the section we introduce edgewise measures and use them to discuss the connection between similarity, complementarity and structural equivalence.

### Structural similarity

**Figure 2:**
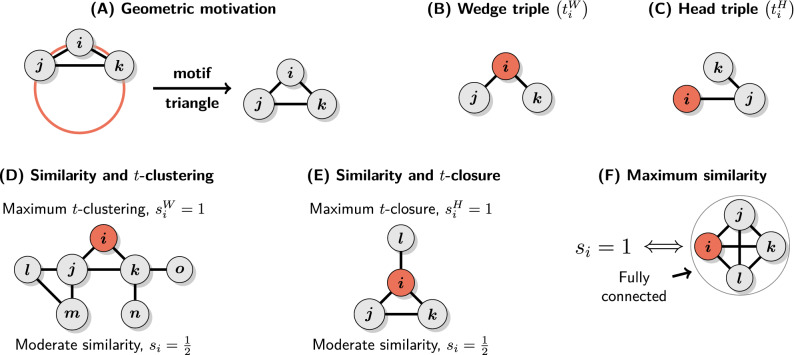
Geometric motivation and the main properties of structural similarity coefficients. (**A**) Metric structure induced by similarity implies transitivity of relations and the abundance of triangles. (**B**, **C**) Wedge and head triples. (**D**) Local clustering can be maximized even when neighbors of the focal node are very differently embedded within the network, while $s_i$ is sensitive to this kind of non-transitivity. (**E**) Local closure can be maximized even for nodes with sparse 1-hop neighborhoods if they are star-like as neighbors with degree one do not generate any head triples. On the other hand, $s_i$ is sensitive to this violation of transitivity. (**F**) Necessary and sufficient conditions for maximum structural similarity.

It is common to think about similarity in terms of distance between different objects in a feature space. Hence, the motivating geometric model for similarity-driven relations posits that nodes are positioned in some metric space and the probability of observing a link between them is a decreasing function of the corresponding distance. Such a generic model can be seen as an instance of the class of Random Geometric Graphs (RGG)^6,12^. The crux is that this very general formulation is enough to guarantee the abundance of triangles (3-cycles) (see Fig. 2A).

Thus, a good starting point for our endeavor is *local clustering coefficient*^28^, of which value for a node *i* will be denoted by $s^W_i$. It is a classical network measure of the density of the 1-hop neighborhood (ego-network) of *i* and is defined as:

2 $$\begin{aligned} s^W_i = \frac{2T_i}{t^W_i} = \frac{\sum _{j,k}a_{ij}a_{ik}a_{jk}}{d_i(d_i-1)} \end{aligned}$$

where $T_i$ is the number of triangles including *i* and $t^W_i$ is the number of wedge triples centered at *i* or 2-paths with *i* in the middle (Fig. 2B). Crucially, $s^W_i \in [0, 1]$ and is equal to 1 if and only if ${\mathscr {N}}_1(i)$ forms a fully connected network. In sociological terms, it measures the extent to which *my friends are friends with each other*. Note, however, that this is only one side of the triadic closure process as it corresponds to the closing of the loop between friends of the focal node *i*. The other part is about the loop between *i* and friends of its friends and local clustering coefficient does not capture it.

To address this issue an alternative *local closure coefficient*^29^ has been proposed more recently:

3 $$\begin{aligned} s^H_i = \frac{2T_i}{t^H_i} = \frac{\sum _{j,k}a_{ij}a_{ik}a_{jk}}{\sum _j a_{ij}(d_j-1)} \end{aligned}$$

where $t^H_i$ is the number of head triples originating from *i*, that is, 2-paths starting at *i* (Fig. 2C). It is also in the range of [0, 1] and attains the maximum value if and only if no neighbor of *i* is adjacent to a node which is not already in ${\mathscr {N}}_1(i)$. In other words, when $s^H_i = 1$ a random walker starting at *i* will never leave ${\mathscr {N}}_1(i)$. Thus, local closure coefficient measures the extent to which *friends of my friends are my friends*, that is, it is a measure of triadic closure between the focal node *i* and neighbors of its neighbors. As a result, it captures exactly that what local clustering is blind to. Since the local clustering and closure coefficients are based on triples we will later refer to them as *t*-clustering and *t*-closure respectively.

The two coefficients complement each other, so it is justified to combine them in a single measure. We now propose such a measure which we will call *structural similarity coefficient*:

4 $$\begin{aligned} s_i = \frac{4T_i}{t^W_i + t^H_i} = \frac{t^W_is^W_i + t^H_is^H_i}{t^W_i + t^H_i} \end{aligned}$$

Note that $s_i$ is equal to the fraction of both wedge and head triples including *i* which can be closed to make a triangle. It is also equivalent to a weighted average of $s^W_i$ and $s^H_i$, which implies that $\min (s^W_i, s^H_i) \le s_i \le \max (s^W_i, s^H_i)$. As we show later, this makes $s_i$ a more general descriptor of local structure than $s^W_i$ or $s^H_i$ alone (cf. “Configuration model” section). Moreover, since $s^W_i = 1$ if and only if ${\mathscr {N}}_1(i)$ is fully connected and $s^H_i = 1$ if there are no links leaving ${\mathscr {N}}_1(i)$ then it must be that $s_i = 1$ if and only if *i* belongs to a fully connected network. Figure 2 provides a summary of the motivation and main properties of $s_i$, including examples of when *t*-clustering and *t*-closure coefficients are maximal while structural similarity is only moderate (Fig. 2D,E). Crucially, unlike local clustering and closure, structural similarity is a comprehensive measure of the density of triangles around a node *i* and therefore captures the full spectrum of local structures implied by the transitivity of similarity-driven relations. Moreover, it is defined for all nodes contained within components with at least 3 nodes. This is in contrast to local clustering which is not defined for nodes with $d_i = 1$.

#### Global similarity

From the global perspective both local clustering and local closure lead to the same conclusion that the corresponding global measure is just the fraction of triples that can be closed to make a triangle^29^. This implies that the same quantity is also the proper global measure of the extent to which relations are driven by similarity. In other words, *global similarity coefficient* is equal to the standard global clustering coefficient and can be defined as:

5 $$\begin{aligned} s = \frac{3T}{\sum _i d_i(d_i-1)} \end{aligned}$$

where *T* is the total number of triangles and the denominator counts the number of triples.

Note that it is indeed a reasonable measure of similarity-driven relations as it is maximized only when a network is fully connected, so all nodes are structurally redundant and each can be removed without affecting the overall connectivity.

### Structural complementarity

First, let us consider an intuitive meaning of complementarity. We posit that two objects are complementary when their features are different but in a well-defined synergistic way. As we will see, this additional synergy constraint is crucial. However, before we discuss this further let us note that in the case of similarity an analogous constraint is built-in by design. For any point there is always only one point minimizing the distance (maximizing similarity) and it is the point itself. In other words, any object is most similar to itself. As a result, there is a well-defined notion of maximal similarity.

On the other hand, the case of difference is more complicated. To make our argument more concrete, let the feature space be ${\mathbb {R}}^k$ with $k \ge 1$. Now, it is easy to see that for any two points *p* and *r* at a distance *d*(*p*, *r*) we can find a third point *s* such that $d(p, s) > d(p, r)$. In other words, for any point *p* there is no well-defined point at the maximum distance. Thus, complementarity cannot be defined in terms of arbitrary differences. Intuitively, defining it in terms of a simple unconstrained heterophily inevitably leads to the conclusion that for any object there is an infinite variety of more and more complementary (different) objects, which clearly does not map well on the common understanding of the notion of complementarity. Thus, we need a definition with the same property as in the case of similarity, that is, one yielding a sequence of ever smaller sets of more and more complementary elements converging to a single well-defined point in the limit of maximum complementarity.

Note that the above abstract argument can be related to known complementarity-driven systems in a rather straightforward manner. For instance, a key and a lock are complementary not because they are just different in an arbitrary fashion, but because they differ in a very specific way by being structural negatives of each other. Similarly, division of labor in modern societies is based on complex synergies between capabilities of different individuals and organizations.

Thus, we argue that complementarity should be defined in terms of distance maximization but with additional constraints ensuring that for any point in the feature space there is only one point at the maximum distance. This can be achieved in several ways, but to keep things simple we will focus on one particularly straightforward solution.

**Figure 3:**
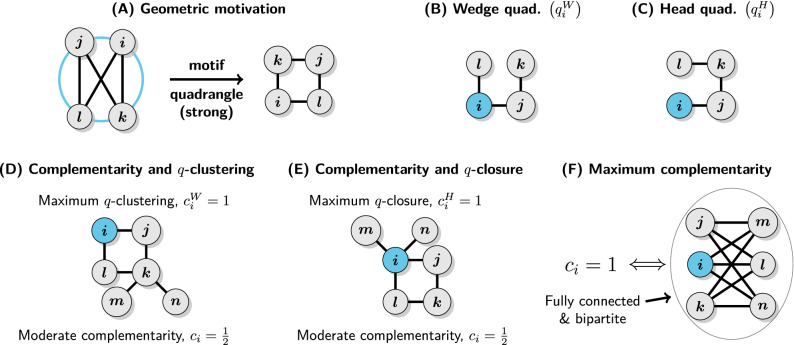
Geometric motivation and the main properties of structural complementarity coefficients. (**A**) On the surface of a (hyper)sphere for each point there is only a single other point at the maximum distance, so complementarity based on distance maximization must lead to the abundance of strong (chordless) quadrangles and locally dense bipartite-like subgraphs. (**B**, **C**) Wedge and head quadruples. (**D**) Local *q*-clustering can be maximized even when some 2-hop neighbors (node *k* on the figure) of the focal node connect to nodes which are not in ${\mathscr {N}}_1(i)$, while the $c_i$ is sensitive to this deviation from the principle of complementarity. (**E**) Local *q*-closure can be maximized even for nodes with sparse 1-hop neighborhoods if they are star-like as neighbors with degree one do not generate any head quadruples. (**F**) Necessary and sufficient conditions for maximum structural complementarity.

We consider nodes as placed on the surface of a *k*-dimensional (hyper)sphere with $k \ge 1$. In this setting for each point there is only a single point at the maximum distance and the maximum distance is the same for all points. Now, if nodes connect preferentially to others who are far away, we obtain a model analogous to similarity, but the connections of a node are not concentrated in its vicinity but instead on the other side of the space. From this it follows that any two connected nodes *i* and *j* will not share a lot of neighbors, so triangles will be rare, but instead the 1-hop neighborhood of *i* should be approximately equal to the 2-hop neighborhood of *j* and *vice versa*, that is, ${\mathscr {N}}_1(i) \approx {\mathscr {N}}_2(j)$ and ${\mathscr {N}}_2(i) \approx {\mathscr {N}}_1(j)$. Such a spatial structure inevitably leads to the abundance of quadrangles (4-cycles) and the presence locally dense bipartite-like subgraphs (Fig. 3A). There are, of course, alternative and more general ways in which geometric models of complementarity-driven relations can be defined (see Ref.^31^ for an excellent example), but distance maximization on a sphere provides a good minimal model highlighting the connection between complementarity, bipartivity and quadrangles.

Depending on the context different authors may refer to slightly different objects when using the term *quadrangle*. Namely, a quadrangle may contain up to two chords or diagonal links. Here we will consider only quadrangles without any chords, which we will call *strong quadrangles*. This choice follows, of course, from the proposed geometric model and the fact that only strong quadrangles are characteristic for dense bipartite-like graphs, which should not have many odd cycles.

Now we can start defining coefficients measuring relations driven by complementarity. As previously, we begin with a local clustering coefficient, which will be called *q*-clustering. It is defined analogously, but this time in terms of quadrangles and wedge quadruples, that is, 3-paths with the focal node *i* at the second position (Fig. 3B):

6 $$\begin{aligned} c^W_i = \frac{2Q_i}{q^W_i} = \frac{\sum _{j \ne i}a_{ij}\sum _{k \ne i, j}a_{ik}(1-a_{jk})\sum _{l \ne i,j,k}a_{kl}a_{jl}(1-a_{il})}{\sum _j a_{ij}[(d_i-1)(d_j-1)-n_{ij}]} \end{aligned}$$

where $Q_i$ is the number of strong quadrangles incident to the focal node *i* and $q^W_i$ is the number of wedge quadruples it belongs to. Note that we consider only quadruples with *i* at the second position, such as (*l*, *i*, *j*, *k*) but not (*k*, *j*, *i*, *l*), in order to avoid double counting and make the number of wedge and head quadruples per quadrangle equal. Intuitively, it quantifies the extent to which the local environment of *i* is bipartite-like and its neighbors are structurally equivalent to each other.

Local *q*-closure coefficient is defined in the same way as the fraction of head quadruples originating from *i* (Fig. 3C) that can be closed to make a (strong) quadrangle:

7 $$\begin{aligned} c^H_i = \frac{2Q_i}{q^H_i} = \frac{\sum _{j \ne i}a_{ij}\sum _{k \ne i, j}a_{ik}(1-a_{jk})\sum _{l \ne i,j,k}a_{kl}a_{jl}(1-a_{il})}{\sum _{j \ne i}a_{ij}\sum _{k \ne i, j}a_{jk}(d_k - 1 - a_{ik})} \end{aligned}$$

where $q^H_i$ is the number of head quadruples starting at *i*. Conceptually, it measures the extent to which the local environment of *i* is bipartite-like and *i* is structurally equivalent to its 2-hop neighbors.

We can now define *structural complementarity coefficient* as the fraction of quadruples including the focal node *i* which can be closed to make a (strong) quadrangle which is equivalent to a weighted average of *q*-clustering and *q*-closure:

8 $$\begin{aligned} c_i = \frac{4Q_i}{q^W_i + q^H_i} = \frac{q^W_ic^W_i + q^H_ic^H_i}{q^W_i + q^H_i} \end{aligned}$$

Note that again we have that $\min (c^W_i, c^H_i) \le c_i \le \max (c^W_i, c^H_i)$, so $c_i$ is always bounded between its constitutive clustering and closure coefficients. This implies that $c_i$ is a more general descriptor than $c^W_i$ or $c^H_i$ alone (cf. “Configuration model” section). Moreover, the interpretations of *q*-clustering and *q*-closure jointly imply that $c_i = 1$ if and only if the focal node *i* belongs to a fully connected bipartite network. Figure 3 presents a summary of the most important terms and facts related to $c_i$.

The geometric model underlying the definition of $c_i$ indeed justifies the interpretation in terms of complementarity or synergy. Nodes are more likely to be connected when they are far away in the feature space, meaning that they have different properties which can be possibly combined in a synergistic manner. Crucially, the mesoscopic network structure that is implied by this model is also related to complementarity in a straightforward manner. Bipartite networks are representations of complementarity-driven systems *par excellence* as they consist of two types of nodes and allow only for connections between them. Thus, $c_i$, being a measure of local bipartivity, is indicative of the degree to which the local environment of a node resembles such a complementarity-driven system.

However, our measure of structural complementarity, while closely related to measures of network bipartivity^27,32^, is also different in at least two important respects. Firstly, unlike bipartivity measures, structural complementarity captures both local bipartivity and density. This is important because even a high degree of bipartivity alone is not a signature of complementarity, since random tree-like structures are also relatively bipartite-like (as evident in Fig.﻿ 3a in Ref.^27^ where bipartivity coefficients, $b_1$ and $b_2$, are much higher than the minimal value of 1/2 even for networks with very low values of $r_1$ parameter which are effectively Erdős–Rényi random graphs). Secondly, bipartivity measures are typically global^27,32^, while structural complementarity coefficients can be defined for edges, nodes and entire graphs (we note, however, that spectral bipartivity can be defined also for individual nodes^33^).

Furthermore, structural complementarity coefficient follows closely the definitions of *i-quad* and *o-quad* coefficients proposed in Ref.^19^. However, it also differs in two important respects. Firstly, it combines both the perspective of wedge (*i-quad*) and head (*o-quad*) quadruples. As we show later (“Configuration model” section), this makes $c_i$ a more general descriptor of local structure and the density of quadrangles, even if for some specific research questions clustering or closure (*i-quad* or *o-quad*) coefficients may still be more appropriate. Secondly, it is based on the notion of strong (chordless) quadrangles instead of the weaker notion allowing for any number of chordal edges. This is necessary for ensuring the direct connection to bipartivity. However, it comes at a cost of making structural complementarity coefficient more sensitive to noise (as strong quadrangles can be easily destroyed by a single erroneous chordal edge) and less capable of detecting structures deviating from the strict assumption of local bipartivity. Of course, $c_i$ can be redefined using weak quadrangles, which would lead to a measure equivalent to a weighted average of *i-quad* and *o-quad* coefficients. However, developing a proper interpretation of weak quadrangles *vis-à-vis* the principles of similarity and complementarity would require a non-negligible amount of additional theoretical and mathematical work, which is outside the scope of this paper. Nonetheless, weak quadrangles may have some interesting applications as, for instance, they seem to be connected to the theory of large quasirandom graphs, of which structure is determined by the amount of general 4-cycles^34^. Thus, we plan to address this problem in the future.

When applied to bipartite networks the quadrangle-based measures can be seen as a generalization of the bipartite clustering coefficient(s)^35,36^. However, the crux is that our structural complementarity coefficients can be applied to unipartite networks in order to quantify jointly local bipartivity and density, which together are indicative of complementarity-driven relations.

#### Global complementarity coefficient

From the global perspective of an entire network there is of course no difference between wedge and head quadruples. Hence, the global coefficient can be defined simply as:

9 $$\begin{aligned} c = \frac{4Q}{\sum _{i,j} a_{ij}[(d_i - 1)(d_j - 1) - n_{ij}]} \end{aligned}$$

where $(i, j) \in E$ and *Q* is the total number of quadrangles with no chords. The denominator counts the total number of quadruples. Note that $c = 1$ if and only if the graph as such is fully connected and bipartite. This agrees with the intuition as this is exactly the structure one should expect in a system composed of two classes of elements in which each element in one class is perfectly complementary to every element in the other.

### Edgewise measures and structural equivalence

#### Similarity

**Figure 4:**
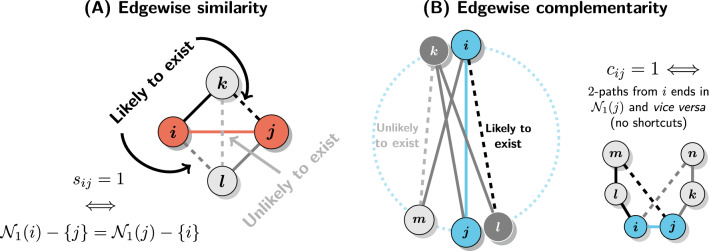
Interpretation of the edgewise structural coefficients. Focal edges are marked with colors (red or blue) and paths starting from the focal (*i*, *j*) edge are black and dark grey. Solid lines denote paths (triples or quadruples) and dashed lines correspond to closing edges that form triangles or quadrangles. (**A**) Logic of edgewise similarity and the necessary and sufficient conditions for maximum $s_{ij}$. If (*i*, *j*) edge is driven by similarity then any neighbor of either *i* or *j* (*k* and *l* on the figure) should be near *i* (or *j*) in the latent space making it likely that it links to the other member of the (*i*, *j*) pair too. On the other hand, *k* and *l* may still be quite far away so the link between them is unlikely to exist. (**B**) Logic of edgewise complementarity and necessary and sufficient conditions for maximum $c_{ij}$. If (*i*, *j*) edge is driven by complementarity then any 2-hop neighbor of *j* (*i*), such as *l* (*k*) on the figure, should be a 1-hop neighbor of *i* (*j*). The quadruple (*i*, *j*, *k*, *l*) corresponds to such a situation. On the other hand, any pair of neighbors of *i* and *j* correspondingly may be located in the latent space close enough to each other as to make a tie between them unlikely (as it happens for nodes *m* and *k* on the figure).

Edgewise structural similarity coefficient is equal to the ratio of triangles including nodes *i* and *j* and the total number of 2-paths traversing the (*i*, *j*) edge (Fig. 4A). In other words, it is equivalent to the number of shared neighbors relative to the total number of neighbors of *i* and *j*, excluding *i* and *j* themselves:

10 $$\begin{aligned} s_{ij} = \frac{2T_{ij}}{t^W_{ij} + t^H_{ij}} = \frac{2n_{ij}}{d_i + d_j - 2} \end{aligned}$$

where $T_{ij}$ is the number of triangles including *i* and *j*, $t^W_{ij}$ is the number of (*k*, *i*, *j*) and $t^H_{ij}$ of (*i*, *j*, *k*) triples. Importantly, $s_{ij}$ is symmetric since $T_{ij} = T_{ji}$ and $t^W_{ij} = t^H_{ji}$.

Note that $s_{ij}$ is closely related to Hamming similarity defined in Eq. (1) and differs only in the $-2$ term in the denominator which accounts for the fact that *i* and *j* are known to be connected. Together with the fact that nodewise coefficient $s_i$ is a weighted average of the corresponding edgewise coefficients, or $\min _j{s_{ij}} \le s_i \le \max _j{s_{ij}}$ for $j \in {\mathscr {N}}_1(i)$, this implies that $s_i$ can be seen as a proxy for the extent to which *i* is structurally equivalent to its own neighbors.

More concretely, it can be shown that:

11 $$\begin{aligned} \min _j H_{ij} < s_i \le \max _j\left( H_{ij}\frac{d_i + d_j}{d_i + d_j - 2}\right) \end{aligned}$$

In other words, high (low) $s_i$ implies the existence of highly (lowly) structurally equivalent neighbor(s). Crucially, this also explains why structural similarity is inherently linked to transitivity. If neighbors of *i* are highly structurally equivalent to it, then it must be likely that if $i \sim j$ and $j \sim k$ then $i \sim k$ or if $i \sim j$ and $i \sim k$ then $j \sim k$. The proof of the above statements is presented in the Supplementary Information (SI: Similarity and structural equivalence).

#### Complementarity

Edgewise structural complementarity coefficient is defined as:

12 $$\begin{aligned} c_{ij} = \frac{2Q_{ij}}{q^W_{ij} + q^H_{ij}} \end{aligned}$$

where $Q_{ij}$ is the number of quadrangles including nodes *i* and *j*, $q^W_{ij}$ is the number of (*j*, *i*, *k*, *l*) and $q^H_{ij}$ of (*i*, *j*, *k*, *l*) quadruples. Again, $Q_{ij} = Q_{ji}$ and $q^W_{ij} = q^H_{ji}$ so $c_{ij}$ is symmetric.

This way $c_{ij}$ can be seen as a joint measure of bipartivity around an (*i*, *j*) edge and structural equivalence between *i* and 1-hop neighbors of *j* and *vice versa*. It measures the extent to which ${\mathscr {N}}_2(i) \approx {\mathscr {N}}_1(j)$ and ${\mathscr {N}}_1(i) \approx {\mathscr {N}}_2(j)$ without requiring dense connections between the 1-hop and 2-hop neighborhoods of *i* and *j*. This is in analogy to edgewise similarity which measures only the extent to which ${\mathscr {N}}_1(i) \approx {\mathscr {N}}_1(j)$ without considering the density of connections between the neighbors of *i* and *j* as this would be a higher-order property unrelated to whether an edge is driven by similarity or not (see Fig. 4 for details).

The connection to structural equivalence is slightly more complicated in the case of complementarity and necessitates an introduction of an additional quantity. For a connected triple (*k*, *i*, *j*) we define *Asymmetric Excess Sørenson Index*:

13 $$\begin{aligned} H_{kj|i} = \frac{n_{jk}-1}{d_k - 1 - a_{jk}} \end{aligned}$$

which measures how many of the connections of *k* are also shared by *j* while disregarding edges (*i*, *k*), (*i*, *j*) and (*j*, *k*). Note that the excess degree of *k* is used in the denominator as the (*i*, *k*) link needs to be ignored. Moreover, $a_{jk}$ term accounts for the possible presence of the (*j*, *k*) link. Finally, 1 is subtracted from $n_{jk}$ to account for the fact that *i* is a shared neighbor of *j* and *k*.

Now, using the fact that $c_i$ is a weighted average of $c_{ij}$’s, or $\min _j c_{ij} \le c_i \le \max _j c_{ij}$, it can be shown that:

14 $$\begin{aligned} 0 \le c_i \le \max _{j, k, l}\left( H_{kj|i}, H_{li|j}\right) \end{aligned}$$

where $j \in {\mathscr {N}}_1(i)$, $k \in {\mathscr {N}}_1(i)-\{j\}$ and $l \in {\mathscr {N}}_1(j)-\{i\}$ (see the proof in SI: Complementarity and structural equivalence).

In other words, $c_i$ is bounded from above by the maximum Asymmetric Excess Sørenson Index between any two of its neighbors or itself and any neighbor of its neighbors. Intuitively, high complementarity can exist only in the presence of high structural equivalence between neighbors of *i* as well as *i* and neighbors of its neighbors.

Crucially, this explains in what sense complementarity-driven relations are not transitive but yet localized. The principle of complementarity enforces both the lack of connections between 1-hop neighbors of *i* as well as a degree of structural equivalence between them. This in turn induces a particular kind of correlations between the connections of *i* and its 1- and 2-hop neighbors which at the same time do not imply transitivity of relations.

## Results

Here we present the results of four case studies analyzing the behavior of structural coefficients in random graph models and using them to answer specific research questions based on several empirical datasets.

### Structural coefficients in random graphs

#### Erdős–Rényi model

In the Erdős–Rényi (ER) model^37^ the expected global similarity, which is of course equivalent to global clustering, is simply ${\mathbb {E}}[s] = p$, or equal to the probability that any edge exists. This is a standard result that follows from the fact that for any (*i*, *j*, *k*) triple the closing (*i*, *k*) edge always exists with probability *p*^15^.

We can use a similar argument to derive the expected value of global complementarity coefficient in the ER model. Let (*i*, *j*, *k*, *l*) be any connected quadruple. It forms a quadrangle with no chords if and only if the (*i*, *l*) edge exists while the (*i*, *k*) and (*j*, *l*) edges do not. Since all edges in the ER model exist independently with probability *p* it means that the expected value of global complementarity coefficient is ${\mathbb {E}}[c] = p(1-p)^2$. Crucially, this result implies that global complementarity decays asymptotically towards 0 in sparse random graphs ($\lim _{n \rightarrow \infty } \langle d_i\rangle /n \rightarrow 0$). This distinguishes it from global bipartivity measures which attain non-minimal values for ER random graphs (cf. Fig. 3a in Ref.^27^).

#### Configuration model

A classical null model for studying nodewise coefficients and their correlations with node degrees is the configuration model in which a particular degree sequence is enforced while apart from that connections are established as randomly as possible^15^. In order to describe the qualitative behavior of the nodewise structural similarity and complementarity we will use the fact that in both cases they are bounded by their corresponding clustering and closure coefficients.

First, note that it is usually conjectured that *t*-clustering should generally decrease with node degree^15^. More recently, it was analytically proven for the family of random networks with power law degree distributions that *t*-clustering is on average roughly constant for low-degree nodes and then starts to decrease more quickly as node degree grows^38^.

On the other hand, it has been shown that local closure coefficient, or *t*-closure in our terminology, is positively correlated with node degree in the configuration model^29^. Thus, these two results together imply that structural similarity $s_i$ can display rich, also non-monotonic, correlations with node degrees depending on the structure of a particular network.

We leave analytical study of the analogous properties of *q*-clustering and *q*-closure for future work. However, since both types of clustering and closure coefficients are based on either wedge or head triples/quadruples and therefore are very similar by construction, we conjecture that they should display the same qualitative behavior in the configuration model. Namely, we expect that *q*-clustering should decrease with node degree, especially for well-connected nodes, and *q*-closure should increase with node degree. As a result, we also expect that structural complementarity should vary with respect to node degree in various, also non-monotonic, ways.

**Figure 5:**
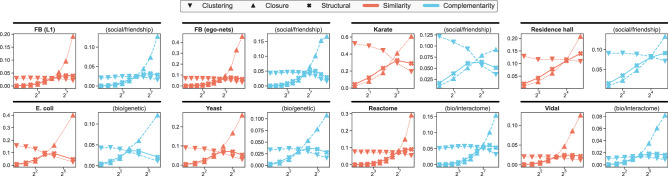
Correlations of clustering, closure and structural coefficients with node degrees in configuration model ensembles based on degree sequences from 8 real-world social and biological networks. See the SI (Fig. S1) for full results based on 28 networks and the description of the datasets. Null distributions of the coefficients were approximated based on 100 samples from Undirected Binary Configuration Model (UBCM). The plots show values averaged for different node degrees in logarithmic bins (base 2). As evident in the figure, structural (similarity and complementarity) coefficients are always bounded between their corresponding clustering and closure coefficients. Furthermore, in all networks clustering coefficients tend to decrease for high degree nodes while closure coefficients grow with respect to degree.

Indeed, our theoretical expectations agree with average trends observed in randomized networks sampled from Undirected Binary Configuration Model^39^ (UBCM; see “Materials and methods” section) fitted to degree sequences of 28 real-world networks. See Fig. 5 for details. The results have two important practical implications. Firstly, structural coefficients often tend to follow closure coefficients more closely for low-degree nodes and clustering coefficients for high degree nodes. In other words, in the configuration model local structure around low-degree (high-degree) nodes is dominated by head (wedge) triples/quadruples, that is, clustering/closure coefficients are good descriptors of the density of triangles/quadrangles only for particular subsets of the degree spectrum. More generally, the degree to which they are relevant depends on the relative abundances of wedge and head paths. On the other hand, structural coefficients are more universal since they are weighted averages of both clustering and closure coefficients with weights reflecting the relative dominance of wedge or head paths.

Secondly, structural coefficients depend on node degrees even in random graphs and therefore, when comparing different networks, their values should be calibrated based on a plausible null model such as UBCM to account for the effects induced purely by the first-order structure (degree sequences).

### Structural coefficients in real networks

**Figure 6:**
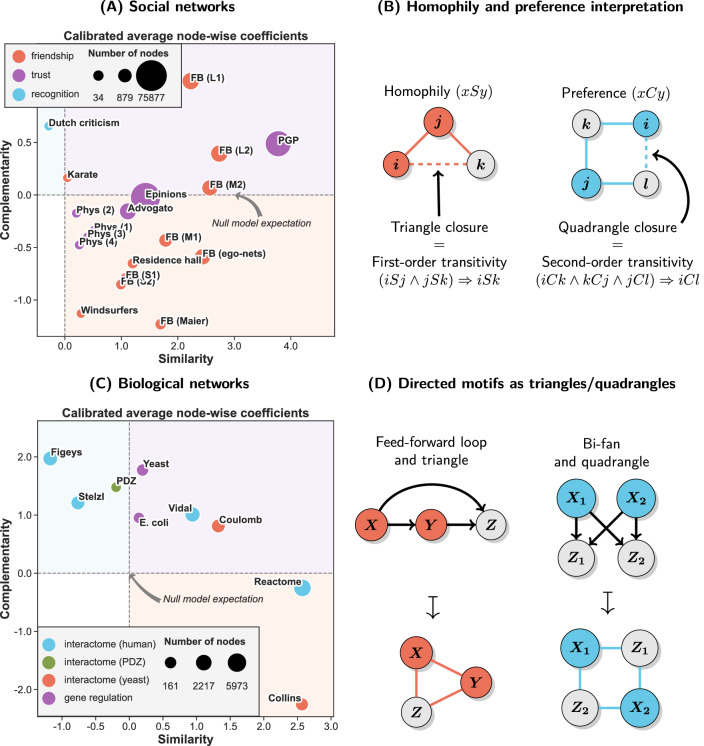
Structural similarity and complementarity in social (19 cases) and biological (9 cases) networks. Scatterplots show calibrated average nodewise coefficients with dashed lines indicating null model expectations based on UBCM (see “Materials and methods” section for details on the datasets and calibration). Colors of the quadrants of the plots indicate configurations of increased values of similarity and complementarity coefficients—high and low (red), low and high (blue), both high (violet). (**A**) Social networks. Almost all feature high similarity and some also increased complementarity (these are mostly large online social networks). The only case with low similarity and relatively high complementarity is the network of Dutch literary criticism representing relationships of recognition (mentioning other’s work, positively or negatively, in an essay or an interview) within a set of notable literary authors^40^. (**B**) Interpretation of similarity and complementarity in terms of homophily, preferences and transitivity. Some social relations, especially those depending on close bonds such as friendship or trust, are often driven by homophily^8,9^. This implies transitivity of ties and the abundance of triangles due to triangle closure. However, other relations such as recognition or skill-based collaboration^23^ are based on preferences decoupled from the properties of the ego. In this case two nodes with similar preferences connect to the same neighbors but not necessarily to each other. This leads to what can be called second-order transitivity, which in turn implies quadrangle closure. (**C**) Biological networks. Most of them feature both increased similarity and complementarity indicating higher structural diversity than in the case of social networks. This is consistent with multiple results reporting the abundance of both triangle and quadrangle based motifs^1–3^. (**D**) Examples of the relationship between directed motifs often reported for biological networks and undirected motifs used for defining structural similarity and complementarity.

We studied structural similarity and complementarity in multiple real-world social and biological networks measuring different kinds of relations—friendship, trust and recognition for social networks as well as gene transcription regulation and general protein-protein interactions (interactomes) for biological networks (see Fig. 6 for details). The goal was to see whether structural similarity and complementarity can be related to some meaningful domain-specific properties of different types of networks.

Our results show that similarity and complementarity in social networks are indeed related to different types of relations. In particular, similarity is stronger in systems driven by homophily, that is, preference for connecting to others who are similar to us, which leads to the transitivity of relations. The importance of similarity seems to be particularly strong for relations depending on close ties such as friendship or trust. This is consistent with decades of research on social networks^8–10,41^. On the other hand, it seems that complementarity plays an important role in shaping of relations in which preferences are decoupled from the properties of the ego, such as recognition (e.g. of value or importance of others), skill-based collaboration^23^ or trade/business interactions^17^. In this case two agents with similar preferences should typically connect to the same neighbors (and therefore be structurally equivalent) but not necessarily to each other, as the preferences of an agent do not have to match its intrinsic properties. This leads to the abundance of quadrangles and the presence of locally dense bipartite-like subgraphs, that is, the structural signatures of complementarity. Interestingly, even though such preference-based relations are not directly transitive, they can be considered second-order transitive due to the implied mechanism of quadrangle closure (see Fig. 6B). We put this tentative hypothesis to a more direct and systematic test in the next section (“Similarity and complementarity in social relations”).

Most of the biological networks feature both relatively high similarity and complementarity. This is consistent with multiple results concerning network motifs characteristic for interactomes as well as neural and gene transcription regulatory networks^1–3^. Namely, structural similarity is linked to the presence of feed-back and feed-forward loops which, when edge directions are unknown or ignored, explains the abundance of triangles. On the other hand, structural complementarity is connected to motifs such as bi-fan and bi-parallel^1^, which imply the abundance of quadrangles (see Fig. 6D). Importantly, these structural patterns can be linked to meaningful domain-specific complementarities between different subsets of elements of a system. For instance, in gene transcription regulatory networks bipartite-like subgraphs with high density of bi-fan motifs (quadrangles) represent dense overlapping regulons (DOR) or groups of operons regulated by similar combinations of input transcription factors^2^.

Our results also point to important differences between social and biological networks. The former, with some exceptions of course, tend to be dominated by similarity while the latter are more structurally diverse, which probably reflects their heterogeneous functional properties and complex evolutionary history (we study this in more detail in “Structural diversity across the tree of life” section). However, it seems that large online social networks also feature increased complementarity relatively often (see Fig. 6A). Thus, it may be worthwhile to study differences between small and large as well as offline and online social networks in the future. In particular, to our best knowledge it is not yet clear what social processes are responsible for significantly high amounts of quadrangles in large online social networks.

### Similarity and complementarity in social relations

Here we test the hypothesis that social relations based on homophily are linked to structural similarity and those based on preference, recognition or skill-based collaboration to structural complementarity. In other words, here we assess the theoretical validity of our approach. For this purpose, we used a set of 34 social networks collected in 17 rural villages in Mayuge District, Uganda^42^. For each village two networks of relations between households were measured: (1) a friendship network and (2) a health advice network (see “Materials and methods” section for details).

This dataset has the structure of a natural experiment as for each village we have two different networks representing relations between the same households in the same period of time which were measured by the same research team(s) using the same method. Thus, they are very likely to be equivalent with respect to any possible covariate except for the type of relation that was measured (friendship or health advice). In other words, they can be compared to each other as nearly perfect synthetic controls^43^ and therefore allow reliable estimation of the effects specific for friendship and health advice relations.

Thus, the dataset provides a perfect setting for testing our hypothesis. Namely, it is sociologically justified to expect the friendship networks to feature high structural similarity as it is a well documented fact that friendship relations are to a large extent shaped by homophily^8–10^. On the other hand, health advice networks should be at least partially driven by complementarity, as the act of advice is usually based on the recognition of and preference for one’s knowledge as well as an information differential between an adviser and an advisee. In other words, advising is based on a synergy between needs and assets of two agents. Moreover, it can be also seen as a particular kind of skill-based collaboration, which is known to be linked to complementarity and heterophily^22,23^. Thus, it is justified to expect the health advice networks to feature high structural complementarity.

**Figure 7:**
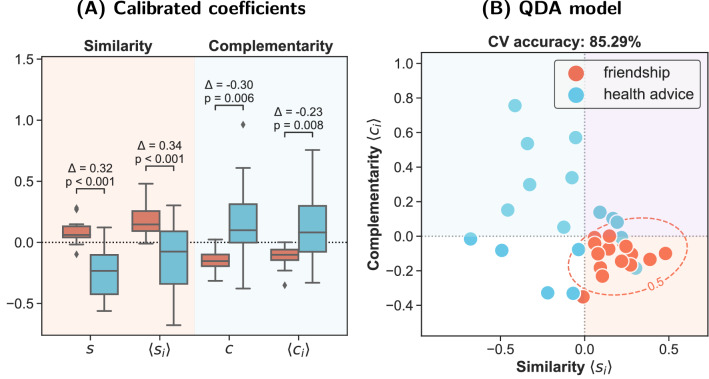
Comparison of structural coefficients between friendship and health advice networks in 17 Ugandan villages. Observed values were calibrated based on 500 samples from UBCM (see “Materials and methods” section). (**A**) Univariate distributions of global and average nodewise coefficients reveal significant differences between the friendship and health advice networks which are consistent with the hypothesis. The friendship networks feature significantly higher structural similarity and the health advice networks higher complementarity. The average differences between networks from the same villages are denoted by $\Delta$’s. Statistical significance was assessed using one-sample *t*-test with two-sided null hypothesis applied to the differences; *p* values were adjusted for multiple testing using Holm–Bonferroni method. (**B**) Bivariate distribution of the calibrated values of the average nodewise similarity and complementarity coefficients. The decision boundary separating the friendship and health advice networks (marked in red) is based on Quadratic Discriminant Analysis (QDA). The out-of-sample classification accuracy was estimated with stratified 17-fold cross-validation (one fold per village).

As evident in Fig. 7A, the results are in clear agreement with the theoretical expectations. The calibrated similarity coefficients (see “Materials and methods” section) in the friendship networks were typically increased relative to the null model (average log-ratios greater than zero) and significantly higher than in the health advice networks ($p < 0.001$). On the other hand, the results for the complementarity coefficients were exactly opposite and in this case the health advice networks featured significantly larger calibrated values ($p < 0.01$).

Thanks to the convenient quasi-experimental structure of the dataset and the calibration accounting for differences in degree sequences the results provide strong support for the claim that, *ceteris paribus*, social relations based on similarity and complementarity leave distinct structural signatures in social networks which can be detected using structural coefficients. In other words, we showed that, all else being equal, similarity-based ties are linked to the abundance of triangles and those based on complementarities to the abundance of quadrangles. This confirms the theoretical validity of the proposed framework and shows that patterns captured by structural coefficients are indeed related to meaningful domain-specific phenomena. Crucially, it also shows that there are types of social relations which are driven not by similarity but complementarity, so the default assumption of homophily is not always adequate.

To gauge the discriminatory power of the coefficients better, we fitted a supervised classifier based on Quadratic Discriminant Analysis (QDA)^44^. To facilitate visualization we used only two predictors: average nodewise similarity and complementarity coefficients. The estimated out-of-sample accuracy was $85.29\%$ (Fig. 7B), which provides further confirmation of the theoretical validity of our approach.

### Structural diversity across the tree of life

Functioning of all biological organisms depends on protein-protein interactions (PPIs), which themselves are constrained by the presence of compatible binding sites^18^. Hence, it can be argued that it is not similar but complementary proteins that are most likely to interact, or that two proteins sharing a neighbor do not have to be connected but instead are likely to share other neighbors (and be structurally equivalent). This view is supported by the statistical over-representation of quadrangle-based motifs in interactome networks^1,2^ as well as recent advances in PPI prediction, which showed that models based on 3-paths (L3) and quadrangle closure outperform those based on 2-paths (L2) and triangle closure^18^. Moreover, there is substantial evidence that protein neighborhoods in interactome networks across the tree of life tend to gradually shift from the dominance of triangles to quadrangles during evolution^45^. Nonetheless, triangle-based motifs are also prevalent in PPI networks and their presence tend to even correlate positively with the abundance of quadrangles^3^. Here we study this problem from the perspective of structural similarity and complementarity and show that increasing complexity of organisms is associated with higher structural diversity of PPI networks, meaning that protein neighborhoods tend to feature increasing numbers of both triangles and quadrangles.

**Figure 8:**
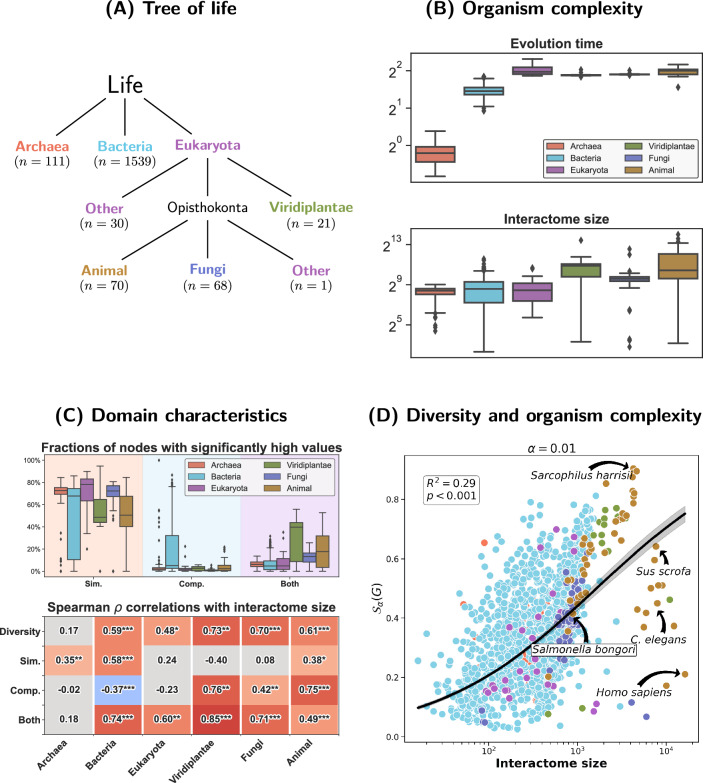
Structural diversity of 1840 interactomes across the tree of life^45^ (see “Materials and methods” section). The analysis was based on proportions of nodes with significantly high values ($$p \le \alpha = 0.01$$) of structural coefficients $$s_i$$ and $$c_i$$ or both of them. We also conducted a sensitivity analysis for alternative levels $$\alpha = 0.05, 0.10$$ (SI: Structural diversity analysis). Significance was based on null distributions estimated with 100 samples from UBCM. (**A**) We followed the division into the domains of Archaea, Bacteria and Eukaryota^46^, but also distinguished three arguably most complex taxa within eukaryotes: green plants (Viridiplantae), fungi and animals (Metazoa). The two “other” classes consist of all other eukaryotes. (**B**) Evolution time and interactome sizes in groups. (**C**) Proportions of nodes with significant values of $$s_i$$ are high in all groups, but tend to be lower among bacteria, which are the only group that feature relatively large numbers of proteins with high structural complementarity. Crucially, it is the more complex eukaryotes, particularly animals and plants, which feature many nodes with significantly high values of both $$s_i$$ and $$c_i$$. This suggests that interactomes of more complex organisms tend to be more structurally diverse. The lower panel shows Spearman $$\rho$$ correlations between interactome size and the proportions as well as the diversity index (16); $$^{***}p \le 0.001$$, $${ }^{**}p \le 0.01$$, $${ }^{*}p \le 0.05$$; Holm–Bonferroni correction for multiple testing was used. Notably, in all groups except Archaea the correlations between interactome size and the diversity index as well as the “both” proportion are strongly positive. Moreover, complementarity increases with network size in plants, fungi and animals but decreases in bacteria, which means that in both cases the general trend goes in the direction of greater structural diversity. (**D**) Structural diversity and interactome size. The general correlation is positive and relatively strong.

We studied PPI networks, or interactomes, of 1840 species across the tree of life^45^ (see Fig. 8 for details). We used network size (number of proteins) for a proxy of the biological complexity of an organism, which is arguably justified as on average interactomes of more complex organisms, such as animals or green plants, are markedly larger than those of bacteria or archaea. Moreover, taxa with larger interactomes on average also tend to have longer average evolution times measured in terms of nucleotide substitutions per site (Fig. 8B).

The analysis was focused on the structural diversity of protein neighborhoods in terms of the local abundance of triangles and quadrangles in relation to the organism complexity (interactome size). We quantified the structure at the level of entire networks in terms of fractions of nodes with significantly high values of $s_i$ and $c_i$ coefficients or both of them (see Fig. 8 for details). Moreover, we also combined the fractions in a synthetic index of structural diversity, ${\mathbb {S}}_{\alpha }(G) \in [0, 1]$ (see “Materials and methods” section for details on calculating *p* values and structural diversity) .

Our analysis (see Fig. 8 for details) indicates a large amount of variation between different species and taxa. It suggests that bacteria interactomes tend to be driven by complementarity, and therefore dominated by quadrangles, to a larger extent than those of other organisms. On the other hand, more complex eukaryotes (green plants, fungi and animals) tend to feature nodes with both high structural similarity and complementarity more often, which implies that protein neighborhoods in their interactomes are more heterogeneous and contain both many triangles and quadrangles. Crucially, this intuition is also confirmed by our structural diversity index which correlates positively with organism complexity (interactome size) (Fig. 8D). Apart from the tail composed of species with large PPI networks where the trend seems to bifurcate into two groups of organisms with unexpectedly high and low diversity scores (with some notable outliers such as *Homo sapiens* and *Sarcophilus harrisii*, or Tasmanian devil), the model provides a relatively good representation of the data generating process. We modeled the relationship using a linear model with logit transform applied to the diversity index and log transform to the number of nodes. Thus, the relationship between “odds” of the diversity index and the number of nodes follows a power law, ${\mathbb {S}}_\alpha (G) / (1-{\mathbb {S}}_\alpha (G)) \propto n^{\gamma }$, with $\gamma = 0.48$ (95% CI: [0.45, 0.51]; $p < 0.001$). We discuss additional details and analyses in the SI (Structural diversity analysis). In particular, we study the stability of the results for different choices of $\alpha$ and examine models controlling for the number of publications on different species (to partially correct for publication bias and resulting differences in terms of interactome completeness).

The results suggest a general tendency towards greater structural diversity in PPI networks of more complex organisms. In many cases this implies an increasing prevalence of quadrangles, which is consistent with the results reported in Ref.^45^ as well as the general importance of complementarity of binding sites for protein–protein interactions^18^. It is also consistent with the accounts of gene duplication occurring during evolution, and in particular whole genome duplication events^47,48^, resulting in the creation of pairs of similarly wired proteins, which together may form multiple quadrangles. These are tentative results which needs to be corroborated with more in-depth analyses before they could have a substantial biological interpretation. Nonetheless, the general picture painted by structural coefficients seems to agree with the existing literature on PPI networks, which suggests that the proposed coefficient may be useful for studying biological networks.

Our results also indicate that, despite the likely increasing importance of quadrangles during evolution, triangles are still important, perhaps as a manifestation of feed-back and feed-forward loops, and interactomes often feature many triangles and quadrangles at the same time, which is consistent with the reports of positive correlations between triangle and quadrangle densities in interactomes^3^. This suggests a way for improving on PPI prediction models based purely on L2 or L3^18^ measures by using a model averaging combining the two metrics by somehow using the information on the local structure provided by structural coefficients. We leave a detailed exploration of this idea for future work.

## Discussion

Starting from first principles based on simple geometric arguments we introduced a framework for measuring similarity- and complementarity-driven relations in networks. We linked both relational principles to their characteristic network motifs—triangles and quadrangles respectively—and defined two general families of structural similarity and complementarity coefficients measuring the extent to which they shape the structure of any unweighted and undirected network. In other words, we showed that both similarity and complementarity leave statistically detectable structural signatures, which opens up new possibilities for studying the structure of various networked systems explicitly in terms of the impact of these two relational principles. We also demonstrated, using multiple empirical examples, that both similarity and complementarity are important for many kinds of social and biological relations. In particular, our results indicate that the customary assumption of homophily may not be appropriate for some social networks, of which structure may be better explained by complementarity.

Even though the connection between the structure of networks and the principle of complementarity is still relatively unexplored, our work was informed by existing studies on quadrangle formation^19^, functional structure^17^, geometry of complementarity-driven networks^31^ and complementarity-based link prediction^18^. It extends this branch of the literature by introducing a set of general graph-theoretical coefficients measuring the density of quadrangles and proposing a simple, minimalistic geometric model linking the principle of complementarity to quadrangles as its characteristic motif.

Furthermore, in contrast to previous studies using quadrangle-based descriptors of local structure^19^, our approach is focused specifically on strong (chordless) quadrangles (cf. Fig. 3A). This makes it, of course, less general, but at the same time allows making a direct connection between the principle of complementarity and network bipartivity. As a result, our work shows that the principle of complementarity induces structures which are both locally bipartite-like and dense, in the same way as similarity is connected to locally dense unipartite subgraphs. Moreover, the proposed structural complementarity coefficients, which measure both bipartivity and density, may be a useful addition to the existing set of measures of bipartivity^27,32^, which do not consider local density. In particular, they may be potentially very useful in studies on systems with so-called functional structure such as production/trade or PPI networks, which are supposed to be characterized by both relatively high bipartivity and density of quadrangle motifs^17^.

Using structural coefficients applied to a rich empirical material, we confirmed that typically social relations such as friendship or trust are driven by similarity and therefore are transitive and linked to the abundance of triangles. However, we also showed that some types of relations, for instance advice, recognition or skill-based collaboration, are more likely to be driven by complementarity, which leads to markedly different local connectivity structures dominated by quadrangles instead of triangles. Importantly, this indicates that such relations are not directly transitive ($i \sim j \wedge j \sim k \Rightarrow i \sim k$), but instead second-order transitive ($i \sim j \wedge j \sim k \wedge k \sim l \Rightarrow i \sim l$), which implies that the principle of triangle (2-path) closure does not capture the dynamics of such systems very well. Instead, it is quadrangle (3-path) closure which is more adequate, so the default assumption of homophily/triadic closure^9–11^ is not always justified. Thus, our results encourage more nuanced approaches to social network analysis and potentially can be used to design novel, more flexible link prediction methods.

We also confirmed that biological networks such as gene transcription regulatory or general PPI networks are more likely to be driven by complementarity and feature more quadrangles than typical social networks. This is consistent with multiple empirical results^2,3,18,45^ and the general mechanism of protein-protein interactions based on complementarity of binding sites^18^. Using structural coefficients, we demonstrated that interactome networks of more complex organisms across the tree of life tend to be more structurally diverse, meaning that they consist of many proteins with neighborhoods containing significantly high numbers of both triangles and quadrangles. This indicates a large degree of heterogeneity of structure in PPI networks and suggests that recent results showing that protein interaction prediction based on 3-path (L3) closure is more effective than the 2-path (L2) closure rule^18^, could be perhaps further improved by combining the L2 and L3 principles in a way informed by the local structure around a given pair of proteins.

An important limitation of our work is the fact that our methods currently can be applied only to undirected and unweighted networks. However, generalizing them to the weighted case should be rather straightforward, and we plan to address this problem in the future. In particular, it should be possible to define weighted structural coefficients following the approach used for defining weighted clustering coefficient in Ref.^49^. On the other hand, the geometric motivation of structural coefficients is inherently undirected, so it is not immediately clear how directed coefficients should be defined. For now, we leave it as an interesting open problem.

In summary, we showed that both similarity and complementarity are important organizational principles shaping the structure of social and biological networks and can be linked to interpretable, domain-specific phenomena. We proposed a set of coefficients for measuring the extent to which they shape the structure of networks and demonstrated the theoretical validity and practical utility of the proposed framework on a rich empirical material.

## Materials and methods

### Computing structural coefficients

Structural coefficients are based on counting triples and triangles (similarity) as well as quadruples and quadrangles (complementarity). While the first problem is relatively easy and efficient methods for solving it are implemented in many popular libraries for graph analysis, the second problem of counting quadruples and quadrangles is more difficult and corresponding efficient algorithms are not widely known. Here we solve both problems by counting all motifs of interest at the level of individual edges and then aggregate the edgewise counts to nodewise or global counts when necessary. We propose an algorithm which can be seen as a special case of a highly efficient exact graphlet counting method proposed in Ref.^50^. We call it PathCensus algorithm as ultimately it counts different types of paths and cycles. Pseudocode for the algorithm and other computational details are discussed in the SI (Structural coefficients and PathCensus).

### Undirected binary configuration model

We used Undirected Binary Configuration Model (UBCM)^39^ for the calibration and assessment of statistical significance of structural coefficients. UBCM is a variant of the configuration model that induces a maximum entropy probability distribution over undirected and unweighted networks with *n* nodes constrained to have a specific expected degree sequence.

UBCM belongs to the family of Exponential Random Graph Models (ERGM)^51^ which induce maximum entropy distributions over networks satisfying some constraints in expectation. Crucially, it means that such models are fully specified by a set of sufficient statistics^52^ describing the desired constraints. Hence, the maximum entropy distributions they induce are as unbiased as possible with respect to any other property^51^.

### Calibrating values of structural coefficients

In the analyses comparing different networks we calibrated observed values of structural coefficients against UBCM in order to account for effects induced purely by the first-order structure (i.e. degree sequences). Such a calibration may be implemented in many different ways, but all reasonable approaches should yield qualitatively comparable results. We explain our method using an example of a calibration of a graph-level statistic such as average nodewise similarity coefficient, $\langle s_i\rangle$.

First, for an observed network *G* calculate the value of a graph statistic of interest, *x*(*G*). Then, sample *R* randomized replicates $G_i$’s of the observed network from a chosen null model (e.g. UBCM) and calculate $x(G_i)$ for $i = 1, \ldots , R$. Finally, the calibrated value of *x*(*G*) based on *R* samples from the null model is defined as the average log-ratio of the observed value and the randomized values:

15 $$\begin{aligned} {\mathscr {C}}(x, R)(G) = \frac{1}{R}\sum _{i=1}^R \log {\frac{x(G)}{x(G_i)}} \end{aligned}$$

Note that the calibrated values are defined using ratios of *x*(*G*) and $x(G_i)$’s, which are expressed in the same units (e.g. triangles/2-paths) and therefore produce a dimensionless quantity, as required by the logarithmic function^53^.

### Assessing significance of structural coefficients

Statistical significance of nodewise structural coefficients was estimated using simulated null distributions based on *R* samples from UBCM. We used the fact that UBCM is a variant of the class of ERGMs^39^ and therefore the probability distribution it induces is fully determined by a set of sufficient statistics^52^, that is, the expected degree sequence in our case. This implies that null distributions of any statistics for nodes with the same degrees are identical, so such nodes are indistinguishable from the vantage point of the model. Thus, we estimated *p* values according to the following procedure:

1. Sample *R* randomized analogues of an observed network *G* from the probability distribution induced by UBCM.
2. For each graph $G_i$ with $i = 1, \ldots R$ calculate a vector of nodewise statistics such as structural similarity coefficient $s_i$.
3. Group calculated values in buckets defined by unique values of node degrees in the observed network *G*. Nodes in randomized networks are treated as if they had the same degrees as their corresponding nodes in *G*.
4. Calculate quantiles of the distributions in the buckets.
5. Set *p* value for each node to $p = 1-\alpha _{\text {max}}$, where $\alpha _{\text {max}}$ is the maximum quantile lower than the observed value for a given node. In all cases we used one hundred quantiles or percentiles.
6. Adjust *p* values for multiple testing using two-stage False Discovery Rate (FDR) correction proposed by Benjamini, Krieger and Yekutieli (Definition 6 in Ref.^54^).

Note that the above procedure ensures at least *R* observations for each node (and more for those with non-unique degrees) and therefore allows estimation of *p* values with a resolution of at least 0.01 when $R \ge 100$ (1/*R* in general).

### Structural diversity index

Let $p^\alpha _S(G), p^\alpha _C(G), p^\alpha _B(G)$ and $p^\alpha _N(G)$ be respectively proportions of nodes with significantly high values (at $p \le \alpha$) of $s_i$ or $c_i$ coefficients or both of them or neither in a graph *G*. Then, we can define analogous proportions conditioned on the set of nodes with at least one significant value as $p^\alpha _{X \mid N'}(G) = p^\alpha _X(G) / (1 - p^\alpha _N(G))$ for $X = S, C, B$. The conditional proportions define a probability distribution ${\mathscr {P}}^\alpha _G$. Finally, structural diversity index of a graph *G* at a significance level $\alpha$ is defined as:

16 $$\begin{aligned} {\mathbb {S}}_\alpha (G) = \frac{(1 - p^\alpha _N){\mathbb {H}}({\mathscr {P}}^\alpha _G)}{\log _2{3}} \end{aligned}$$

where ${\mathbb {H}}({\mathscr {P}}^\alpha _G) = -\sum _{X}p_X^\alpha (G)\log _2{p_X^\alpha (G)}$ is Shannon entropy functional^55^ and $\log _2{3}$ term in the denominator is a normalizing constant ensuring that ${\mathbb {S}}_\alpha (G) \in [0, 1]$. This measure captures structural heterogeneity of node neighborhoods while being penalized for networks with mostly random-like structure.

### pathcensus package

We implemented all the methods and algorithms for calculating structural coefficients as well as several other utilities including most appropriate null models and auxiliary methods for conducting statistical inference in pathcensus package for Python. The core routines are just-in-time compiled to highly optimized C code using *Numba* library^56^ ensuring high efficiency. The package has an extensive documentation including several usage examples. It is available at *Python Package Index* (https://pypi.org/project/pathcensus) and can be installed as any regular Python package.

## Supplementary Information

Supplementary Information.

## Data Availability

This study did not generate any new data. Networks used in this paper are freely accessible from the Netzschleuder repository: https://networks.skewed.de. Preprocessed data used in the analyses as well as the code needed for reproducing the data and all the analyses are available at GitHub: https://github.com/sztal/scs-paper.
