# Supplementary material for: Structural measures of similarity and complementarity in complex networks
Source: Sci Rep. 2022 Oct 4;12:16580. doi: 10.1038/s41598-022-20710-w (PMC9532398; doi:10.1038/s41598-022-20710-w)
Supplement: Supplementary file 1 — Supplementary Information. [file 41598_2022_20710_MOESM1_ESM.pdf]

Structural measures of similarity and complementarity in complex networks

Szymon Talaga[1,*] **and Andrzej Nowak**[2,3]

[1]Robert Zajonc Institute for Social Studies, University of Warsaw, Stawki 5/7, 00-183 Warsaw, Poland
[2]Faculty of Psychology, University of Warsaw, Stawki 5/7, 00-183 Warsaw, Poland
[3]Department of Psychology, Florida Atlantic University, 777 Glades Rd, Boca Raton, FL 33431, USA
[*]stalaga@uw.edu.pl

Similarity and structural equivalence

Here we derive the relationship between similarity coefficient s_{ij} and s_i and structural equivalence. First, we show that s_i is a weighted average of the edgewise coefficients s_{ij}'s for $j \in \mathcal{N}_1(i)$, that is:

$$s_i = \frac{4T_i}{t_i^W + t_i^H} = \frac{\sum_j \left(t_{ij}^W + t_{ij}^H\right) s_{ij}}{\sum_j t_{ij}^W + t_{ij}^H} \tag{S1}$$

Note that Eq. (5) in the Main Text implies that $\left(t_{ij}^W + t_{ij}^H\right) s_{ij} = 2T_{ij}$. Moreover, since each triangle including i is shared with two other neighbors we have that:

$$\sum_{j \in \mathcal{N}_1(i)} \left(t_{ij}^W + t_{ij}^H\right) s_{ij} = \sum_{j \in \mathcal{N}_1(i)} 2T_{ij} = 4T_i \tag{S2}$$

On the other hand, $t_{ij}^W + t_{ij}^H$ is the number of 2-paths traversing the (i, j) edges so it can be written as $t_{ij}^W + t_{ij}^H = d_i + d_j - 2$. Hence, it is easy to see that:

$$\begin{aligned} \sum_{j \in \mathcal{N}_1(i)} t_{ij}^W + t_{ij}^H &= \sum_{j \in \mathcal{N}_1(i)} (d_i + d_j - 2) \\ &= d_i(d_i - 1) + \sum_{j \in \mathcal{N}_1(i)} (d_j - 1) \\ &= t_i^W + t_i^H \end{aligned} \tag{S3}$$

Finally, substituting (S2) and (S3) into (S1) we confirm the desired equality.

Now, we use a common definition of structural equivalence in terms of Sørenson Index (normalized Hamming similarity) and note its direct connection to our notion of edgewise structural similarity s_{ij}:

$$H_{ij} = \frac{2n_{ij}}{d_i + d_j} = \frac{2T_{ij}}{d_i + d_j} = s_{ij}\frac{d_i + d_j - 2}{d_i + d_j} \tag{S4}$$

The above implies that $H_{ij} < s_{ij}$ for all (i, j) edges for which s_{ij} is defined. And since we established that s_i is a weighted average of s_{ij}'s with $j \in \mathcal{N}_1(i)$ we have that:

$$\min_j H_{ij} < \min_j s_{ij} \leq s_i \leq \max_j s_{ij} = \max_j \left(H_{ij}\frac{d_i + d_j}{d_i + d_j - 2}\right) \tag{S5}$$

Note that for large values of $d_i + d_j$ the above is approximately equivalent to:

$$\min_j H_{ij} < s_i \leq \max_j H_{ij} \tag{S6}$$

In other words, we showed that the similarity coefficient of a node i is approximately bounded between minimum and maximum structural equivalence (Sørenson Index) between itself and any of its neighbors.

Complementarity and structural equivalence

Here we derive the relationship between complementarity coefficients c_{ij} and c_i and structural equivalence. We start by showing that c_i is a weighted average of the edgewise coefficients c_{ij}'s for $j \in \mathcal{N}_1(i)$, that is:

$$c_i = \frac{4Q_{ij}}{q_i^W + q_i^H} = \frac{\sum_j \left(q_{ij}^W + q_{ij}^H\right) c_{ij}}{\sum_j q_{ij}^W + q_{ij}^H} \tag{S7}$$

Using Eq. (11) from the Main Text we can write $2Q_{ij} = \left(q_{ij}^W + q_{ij}^H\right) c_{ij}$. Moreover, each strong quadrangle including a node i is shared with exactly two other neighbors. Hence, we have that:

$$\sum_{j \in \mathcal{N}_1} \left(q_{ij}^W + q_{ij}^H\right) c_{ij} = \sum_{j \in \mathcal{N}_1(i)} 2Q_{ij} = 4Q_i \tag{S8}$$

Next, note that each 3-path starting at an (i, j) edge defines a unique ordered quadruple of the form (i, j, k, l) or (j, i, k, l). The first form is counted as a head quadruple of the node i and a wedge quadruple of the node j and in the second case the order is reversed. And since $q_{ij}^W + q_{ij}^H$ is the number of 3-paths starting at the (i, j) edge it must hold that:

$$\sum_{j \in \mathcal{N}_1(i)} \left(q_{ij}^W + q_{ij}^H\right) = q_i^W + q_i^H \tag{S9}$$

Finally, note that (S8) and (S9) jointly mean that (S7) must be true. As a result, for $j \in \mathcal{N}_1(i)$ we have that:

$$\min_j c_{ij} \leq c_i \leq \max_j c_{ij} \tag{S10}$$

Now, in order to derive the connection between complementarity coefficients and structural equivalence we need first to introduce one additional quantity. For a connected triple (k, i, j) we define Asymmetric Excess Sørenson Index:

$$H_{kj|i} = \frac{n_{jk} - 1}{d_k - 1 - a_{jk}} \tag{S11}$$

which measures how many of the connections of k are also shared by j while disregarding edges (i, k), (i, j) and (j, k).

Next, we also need to use the notion of weak quadrangles allowing for any number of chordal edges. Let $W_{ij} \geq Q_{ij}$ be the number of quadrangles with any number of chords incident to the (i, j) edge. We also define weak edgewise complementarity to be $h_{ij} = W_{ij}/(q_{ij}^W + q_{ij}^H) \geq c_{ij}$. It is easy to see that:

$$W_{ij} = \sum_{k \in \mathcal{N}_1(i) - \{j\}} n_{jk} - 1 \tag{S12}$$

On the other hand, the number of 3-paths starting at the (i, j) edge is:

$$\begin{aligned} q_{ij}^W + q_{ij}^H &= \sum_{k \in \mathcal{N}_1(i) - \{j\}} (d_k - 1) + \sum_{l \in \mathcal{N}_1(j) - \{i\}} (d_l - 1) - 2n_{ij} \\ &= \sum_{k \in \mathcal{N}_1(i) - \{j\}} (d_k - 1 - a_{jk}) + \sum_{l \in \mathcal{N}_1(j) - \{i\}} (d_l - 1 - a_{il}) \end{aligned} \tag{S13}$$

since q_{ij}^W is the number of (j, i, k, l) and q_{ij}^H of (i, j, k, l) quadruples. The second equality comes from the fact that $n_{ij} = \sum_k a_{jk} = \sum_l a_{il}$. Now, we can use (S11), (S12) and (S13) to rewrite the weak edgewise complementarity as:

$$h_{ij} = \frac{\sum_k (d_k - 1 - a_{jk}) H_{kj|i} + \sum_l (d_l - 1 - a_{il}) H_{li|j}}{\sum_k (d_k - 1 - a_{jk}) + \sum_l (d_l - 1 - a_{il})} \tag{S14}$$

As a result, for $k \in \mathcal{N}_1(i) - \{j\}$ and $l \in \mathcal{N}_1(j) - \{i\}$ we have that:

$$\min_{k,l}(H_{kj|i}, H_{li|j}) \leq h_{ij} \leq \max_{k,l}(H_{kj|i}, H_{li|j}) \tag{S15}$$

Using (S10) we can write:

$$\min_{j,k,l}(H_{kj|i}, H_{li|j}) \leq h_i \leq \max_{j,k,l}(H_{kj|i}, H_{li|j}) \tag{S16}$$

Finally, since by definition $c_{ij} \leq h_{ij}$ this implies:

$$0 \leq c_{ij} \leq \max_{k,l}(H_{kj|i}, H_{li|j}) \tag{S17}$$

as well as:

$$0 \leq c_i \leq \max_{j,k,l}\left(H_{kj|i}, H_{li|j}\right) \tag{S18}$$

In other words, we just showed that c_i is bounded from above by the maximum Asymmetric Excess Sørenson Index between any two of its neighbors or itself and any neighbor of its neighbors. Moreover, in the weak case we also have a lower bound of the same nature. We leave a more detailed analysis of the notion of weak complementarity for future work.

Structural coefficients and `PathCensus`

Formulas and algorithm

Edge-level counts of triples, quadruples, triangles and quadrangles are computed with the algorithm S1. Node and global counts can be obtained by aggregating edge counts. The rules of aggregation are summarized in Table S1. Table S2 presents detailed formulas for all structural coefficients expressed in terms of the aggregated counts.

Table S1. Formulas for aggregating from edge to node and global counts

	Counting level		
	Edge	Node	Global
Paths			
Wedge triples	t^W_{ij}	$t^W_i = \sum_j t^W_{ij}$	$t^W = \frac{1}{2}\sum_{i,j} t^W_{ij}$
Head triples	t^H_{ij}	$t^H_i = \sum_j t^H_{ij}$	$t^H = \frac{1}{2}\sum_{i,j} t^H_{ij}$
Wedge quadruples	q^W_{ij}	$q^W_i = \sum_j q^W_{ij}$	$q^W = \frac{1}{2}\sum_{i,j} q^W_{ij}$
Head quadruples	q^H_{ij}	$q^H_i = \sum_j q^H_{ij}$	$q^H = \frac{1}{2}\sum_{i,j} q^H_{ij}$
Cycles			
Triangles	T_{ij}	$T_i = \frac{1}{2}\sum_j T_{ij}$	$T = \frac{1}{6}\sum_{i,j} T_{ij}$
Quadrangles	Q_{ij}	$Q_i = \frac{1}{2}\sum_j Q_{ij}$	$Q = \frac{1}{8}\sum_{i,j} Q_{ij}$

Table S2. Formulas for structural coefficients based on path and cycle counts

		Relational principle	
Level	Coefficient	Similarity	Complementarity
Edges	Structural	$s_{ij} = \frac{2T_{ij}}{t_{ij}^W + t_{ij}^H}$	$c_{ij} = \frac{2Q_{ij}}{q_{ij}^W + q_{ij}^H}$
Nodes	Structural	$s_i = \frac{4T_i}{t_i^W + t_i^H}$	$c_i = \frac{4Q_i}{q_i^W + q_i^H}$
	Clustering	$s_i^W = \frac{2T_i}{t_i^W}$	$c_i^W = \frac{2Q_i}{q_i^W}$
	Closure	$s_i^H = \frac{2T_i}{t_i^H}$	$c_i^H = \frac{2Q_i}{q_i^H}$
Global[1]	Structural	$s = \frac{6T}{t^W + t^H}$	$c = \frac{8Q}{q^W + q^H}$
	Clustering	$s^W = \frac{3T}{t^W}$	$c^W = \frac{4Q}{q^W}$
	Closure	$s^H = \frac{3T}{t^H}$	$c^H = \frac{4Q}{q^H}$

[1]All global measures are equivalent.

Algorithm S1 `PathCensus` algorithm. It takes an undirected graph $G = (V, E)$ with $|V| = n$ and $|E| = m$ as input and returns an array of edgewise counts of wedge and head triples and quadruples as well as triangles and (strong) quadrangles. For better performance E can be defined (without loss of generality) to ensure that for all edges (i, j) it holds that $d_i \leq d_j$.

Initialize empty C ▷ $m \times 8$ array for storing path counts
Initialize R such that $R_i = 0 \quad \forall i \in V$ ▷ $n \times 1$ array for keeping track of node roles
Let D be the degree sequence of G ▷ $n \times 1$ array
Initialize $u = 0$
for $e = (i, j) \in E$ **do** ▷ the loop may be parallelized
    Set $u = u + 1$
    Initialize $T_{ij}, t_{ij}^W, t_{ij}^H = 0$ ▷ counts of triangles and wedge and head triples
    Initialize $Q_{ij}, q_{ij}^W, q_{ij}^H = 0$ ▷ counts of strong quadrangles and wedge and head quadruples
    Initialize $\text{Star}_i, \text{Star}_j, \text{Tri}_{ij} = \emptyset$ ▷ Empty sets for keeping track of nodes with different roles
    for $(k \neq j) \in \mathcal{N}_1(i)$ **do**
        Add k to Star_i and set $R_k = 1$
        Set $t_{ij}^W = t_{ij}^W + 1$
    for $(k \neq i) \in \mathcal{N}_1(j)$ **do**
        if $R_k = 1$ **then**
            $T_{ij} = T_{ij} + 1$
            Remove k from Star_i, add k to Tri_{ij} and set $R_k = 3$
        else
            Add k to Star_j and set $R_k = 2$
        $t_{ij}^H = t_{ij}^H + 1$
    for $k \in \text{Star}_i$ **do** ▷ This internal nested loop determines computational complexity
        for $(l \neq i) \in \mathcal{N}_1(k)$ **do**
            if $R_l = 2$ **then**
                $Q_{ij} = Q_{ij} + 1$
    for $k \in \text{Star}_i$ **do**
        Set $q_{ij}^W = q_{ij}^W + D_k - 1$ and $R_k = 0$
    for $k \in \text{Star}_j$ **do**
        Set $q_{ij}^H = q_{ij}^H + D_k - 1$ and $R_k = 0$
    for $k \in \text{Tri}_{ij}$ **do**
        Set $q_{ij}^W = q_{ij}^W + D_k - 2$
        Set $q_{ij}^H = q_{ij}^H + D_k - 2$
        Set $R_k = 0$
    Set $C_u = \left(T_{ij}, t_{ij}^W, t_{ij}^H, Q_{ij}, q_{ij}^W, q_{ij}^H\right)$ ▷ Set u-th row of C
return C

Calculating counts for reversed edges

Note that in our implementation t_{ij}^{W} counts the number of (k,i,j) and t_{ij}^{H} tracks (i,j,k) triples. Thus, we have that $t_{ij}^{W} = t_{ji}^{H}$. Similarly, q_{ij}^{W} counts (j,i,k,l) and q_{ij}^{H} (i,j,k,l) quadruples, so again we have that $q_{ij}^{W} = q_{ji}^{H}$. On the other hand, counts of triangles and quadrangles are symmetric. As a result, for the purpose of counting we can assume that all edges are of the form $i < j$ and still be able to count everything correctly. In other words there is no need to consider each undirected edge twice.

Computational complexity

It is clear from the structure of the three nested loops that the asymptotic worst-case computational complexity of both algorithms is $O(m\Delta S d_{\max})$ where m is the number of edges, ΔS is the maximum size of a Star$_i$ set (see Algorithm S1) and $d_{\max}$ is the maximum node degree. This agrees with the analysis presented by the authors of the more general graphlet counting method[1], which inspired our `PathCensus` algorithm. However, in practice the runtime can be reduced by enforcing that edges are defined to satisfy the condition $d_i \leq d_j$ (note that this can always be done without loss of generality). The impact of this optimization can be quite significant for networks with highly heterogeneous degree distributions. For instance, in the case of the PGP web of trust network[2] ($n = 39796, \langle d_i \rangle = 9.91, d_{\max} = 1696$) it yields almost 4 times shorter runtime on average.

Degree correlations in configuration model

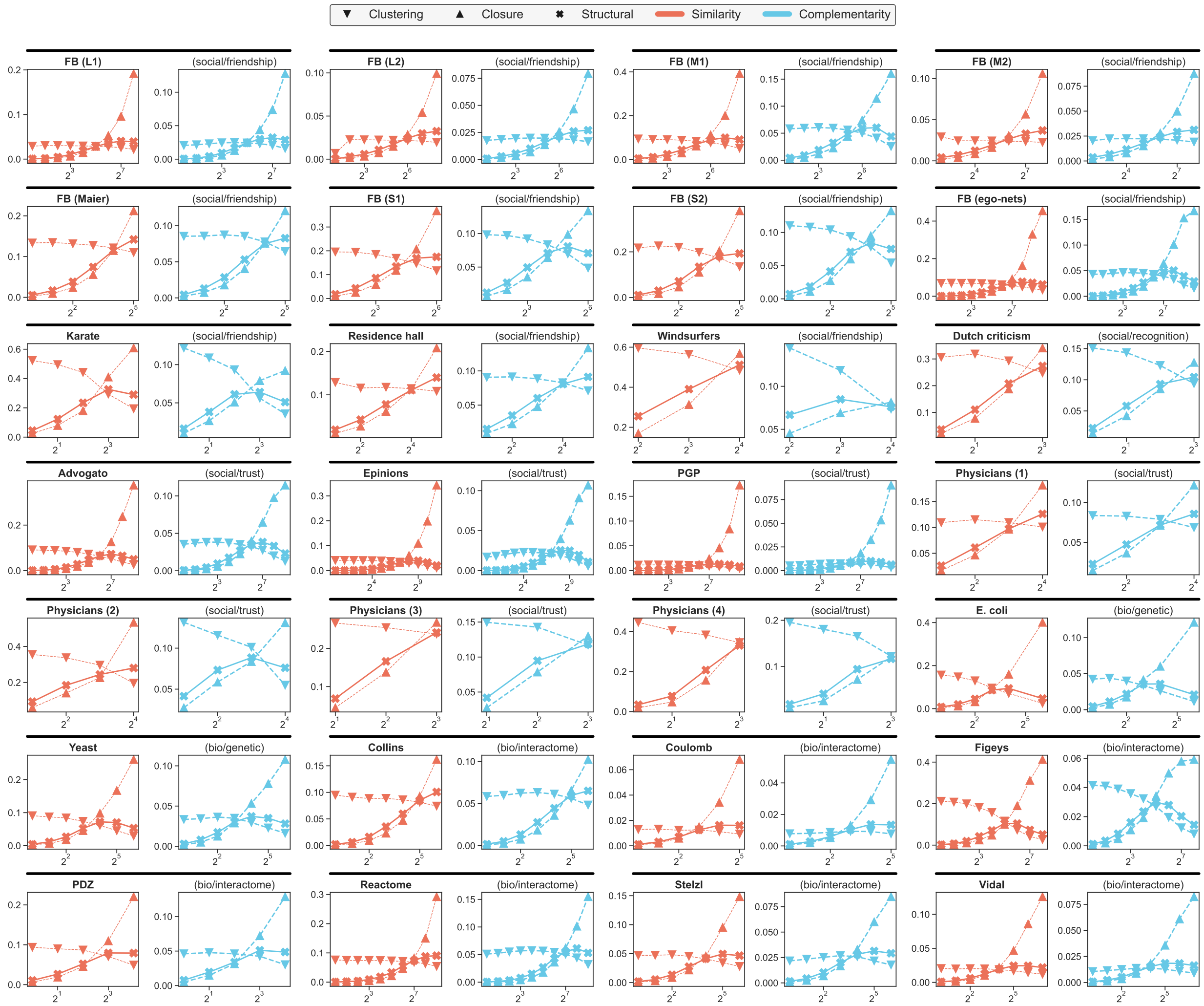

Figure S1. Correlations of clustering, closure and structural coefficients with node degrees in configuration model ensembles based on degree sequences from 28 real-world social and biological networks (see Section: Network datasets). Null distributions of the coefficients were approximated based on 100 samples from Undirected Binary Configuration Model (UBCM). The plots show values averaged for different node degrees in logarithmic bins (base 2).

Structural diversity analysis

Here we provide additional details for the corresponding analysis in the Main Text (Section: Structural diversity across the tree of life). We present results for three different choices of significance level, $\alpha = 0.01, 0.05, 0.10$ used for detecting nodes with high structural similarity and complementarity. We show that qualitative results are stable for all values of α, even though quantitative details change in some cases.

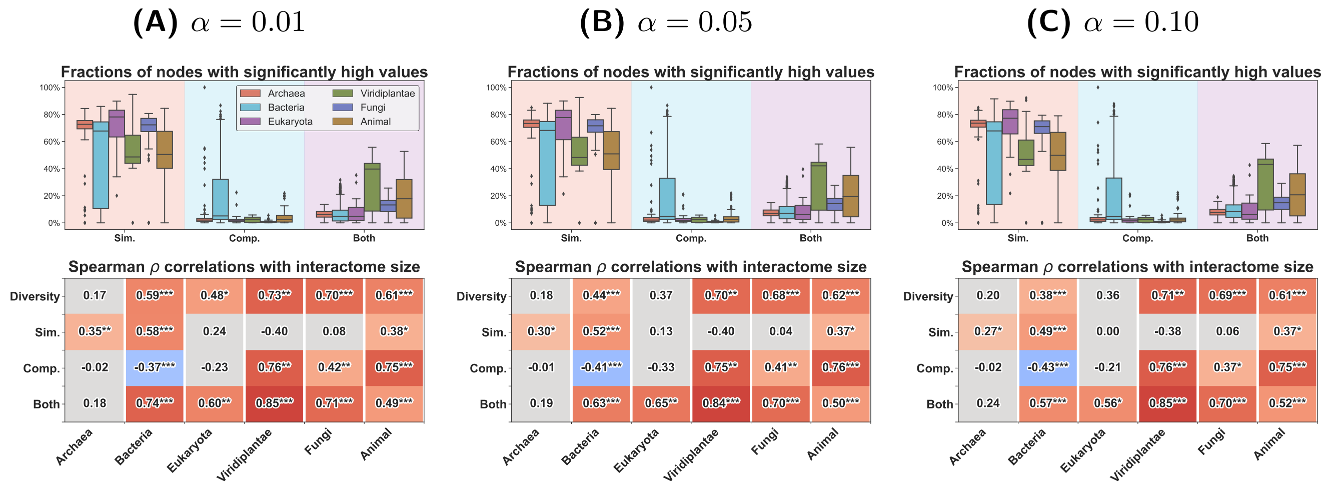

Figure S2. General characteristic of the structure of interactomes in different domains/groups. Apart from some minor details the results for all values of α are the same.

Linear model stability and diagnostics

We described the relationship between structural diversity, $y = \mathbb{S}_\alpha(G)$, defined in Eq. (11) in the Main Text, and interactome size n (number of proteins) using a transformed linear model of the form:

$$\eta(\hat{y}) = c + \gamma \log n \qquad \text{(S19)}$$

where $\eta(x) = x/(1-x)$ is the logit transformation. This parametric form ensured model predictions bounded in $(0,1)$, which was necessary as the structural diversity index ranges from 0 to 1. On the other hand, since the logit transformation is not defined for 0's and 1's we had to drop some part of the observations, namely, 119, 94 and 88 cases for $\alpha = 0.01, 0.05, 0.10$ respectively. These observations corresponded almost exclusively to organisms with small interactomes as indicated by small average numbers of nodes (35.07, 30.98 and 28.15) as compared to the overall average of 544.06 nodes. Moreover, all of them were dropped because of $\mathbb{S}_\alpha(G) = 0$, which is consistent with the hypothesis that less complex organisms tend to have less structurally diverse interactomes.

As evident in Fig. S3 the qualitative trend is the same for all values of α. However, the goodness-of-fit of the model is highest for $\alpha = 0.01$. This is not surprising. Higher values of α correspond to higher type I error rates, meaning that the estimated fractions of nodes with significantly high values of s_i and c_i are more noisy. Table S3 presents estimated parameters and other numerical details.

Moreover, since the accuracy of interactome networks may depend on the extent to which a given species has been studied, we also fitted extended models including the logarithm of the number of publications about a given species as the second predictor. This enabled a partial control for possible biases induced by differences in terms of the incompleteness of the data available for more and less frequently studied organisms. As Table S3 shows, the effects of publication count (b) were insignificant in all cases.

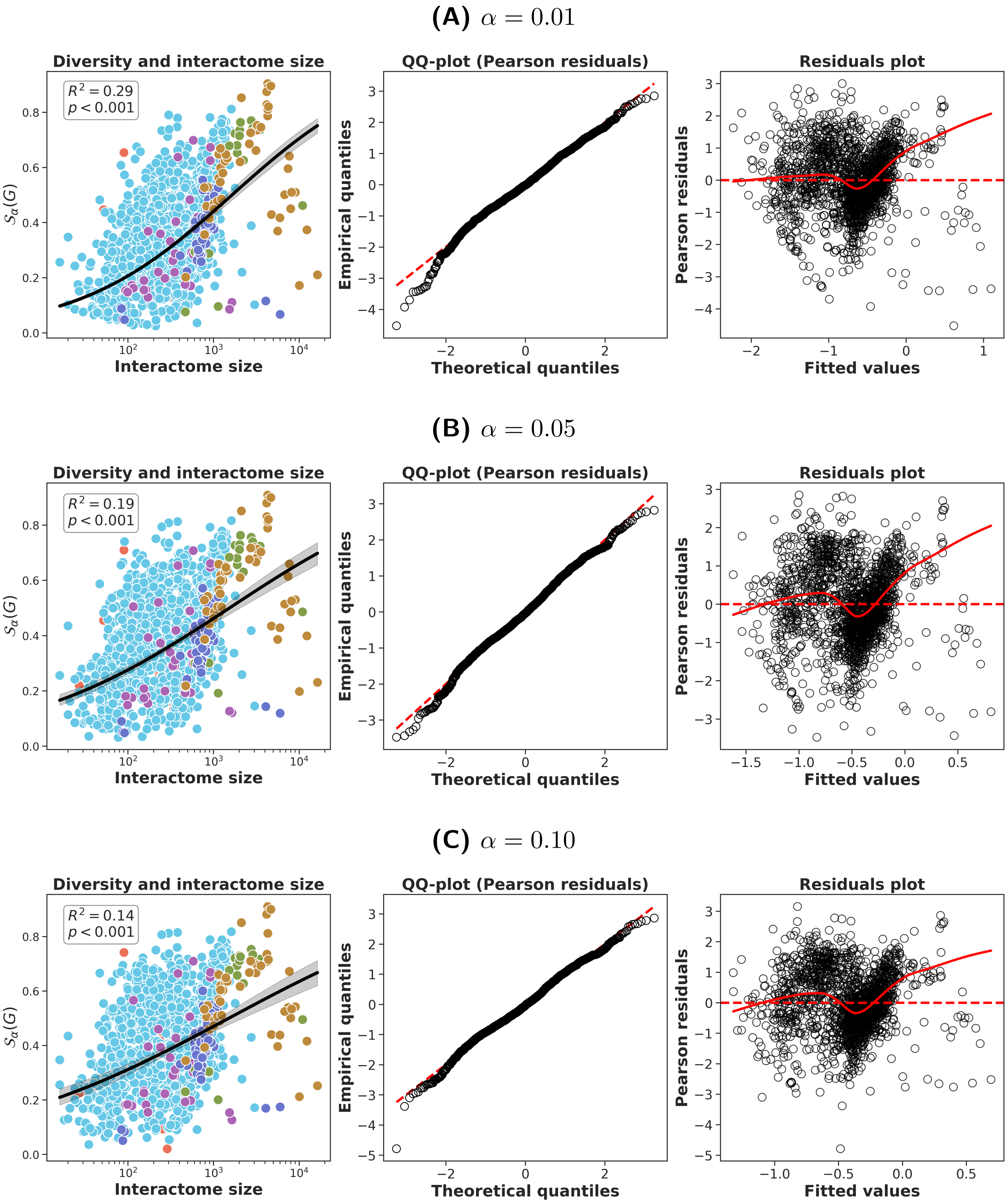

Figure S3. Model fit and residual diagnostics for $\alpha = 0.01, 0.05, 0.10$. In all cases the qualitative trend is similar but R^2 drops for higher values of α. Moreover, in all cases residuals are approximately normal apart from larger fluctuations in the region of high fitted values. This is an effect of the apparent bifurcation of the conditional distribution of structural diversity index for species with large interactomes discussed in the Main Text.

Table S3. Estimated parameters of the linear models[1]

	$\alpha = 0.01$ ($N = 1721$)				$\alpha = 0.05$ ($N = 1746$)				$\alpha = 0.10$ ($N = 1752$)			
	Value	SE	z	p	Value	SE	z	p	Value	SE	z	p
c	-3.562	0.075	-47.476	< 0.001	-2.595	0.133	-19.477	< 0.001	-2.139	0.164	-13.048	< 0.001
γ	0.481	0.015	32.256	< 0.001	0.353	0.023	15.299	< 0.001	0.292	0.027	10.817	< 0.001
	R^2 = 0.290, F(1719,1) = 1040, p < 0.001				R^2 = 0.188, F(1744,1) = 234, p < 0.001				R^2 = 0.290, F(1750,1) = 117, p < 0.001			
	Skew = -0.296, Kurtosis = 3.728 (residuals)				Skew = -0.134, Kurtosis = 3.135 (residuals)				Skew = -0.129, Kurtosis = 3.234 (residuals)			
	**Models accounting for publication count (b)**											
	$\alpha = 0.01$ ($N = 1662$)				$\alpha = 0.05$ ($N = 1685$)				$\alpha = 0.10$ ($N = 1752$)			
	Value	SE	z	p	Value	SE	z	p	Value	SE	z	p
c	-3.563	0.079	-44.918	< 0.001	-2.591	0.143	-18.175	< 0.001	-2.129	0.172	-12.369	< 0.001
γ	0.481	0.017	28.633	< 0.001	0.353	0.026	13.730	< 0.001	0.290	0.029	9.971	< 0.001
b	-0.0003	0.011	-0.026	0.979	0.0014	0.009	-0.155	0.876	0.0005	0.009	0.062	0.951

[1] Standard errors robust to cluster correlation (within taxa) were used.

Network datasets

All network datasets were downloaded from the Netzschleuder repository[3]. In all analyses only largest connected components were used and networks were simplified by removing multilinks and self-loops. Moreover, in the case of directed or weighted networks edge directions and weights were ignored.

Individual network datasets are described below and their unique names within the repository are provided in the following subsection headings. Each dataset can be accessed via a generic link of the form: networks.skewed.de/net/<name> where <name> is a placeholder which should be substituted with the name of a specific dataset.

Below we list datasets in groups corresponding to the three empirical analyses presented in the Main Text. Each section ends with a table with most important descriptive statistics for all networks. The statistics were calculated for the largest connected component.

Networks used in "Structural coefficients in real networks"

Within-organization Facebook friendships (`facebook_organizations`)

Six undirected and unweighted networks of friendships among users on Facebook who indicated employment at one of the target corporations (S1, S2, M1, M2, L1, L2)[4]. Companies range in size from small to large. Only edges between employees at the same company are included in a given snapshot.

Maier Facebook friends (`facebook_friends`)

A small anonymized Facebook ego network (undirected and unweighted), from April 2014[5]. Nodes are Facebook profiles, and an edge exists if the two profiles are "friends" on Facebook.

Facebook ego-network (`ego_social`)

The network is a combination of 10 ego-nets sampled from Facebook[6]. The network is undirected and unweighted.

Zachary Karate Club (`karate`)

Undirected and unweighted network of friendships among members of a university karate club[7]. We used the corrected version with 78 instead of 77 edges.

ANU Residence Hall network (`residence_hall`)

A network of friendships among students living in a residence hall at Australian National University[8]. The original network is directed and weighted with edges indicating that resident i named resident j as a friend, and weight indicating the level of friendship: 5 (best friend), 4 (close friend), 3 (friend), 2, 1.

Windsurfers network (`windsurfers`)

A network of interpersonal contacts among windsurfers in southern California during the Fall of 1986[9]. The original network is weighted with weights indicating the perception of social affiliations majored by the tasks in which each individual was asked to sort cards with other surfer's name in the order of closeness.

Dutch literacy criticism (`dutch_criticism`)

A network of criticisms among Dutch literary authors in 1976[10]. The directed edge (i, j) denotes that an author i passed judgment on author j's work in an interview or review. The original network is also signed and have positive and negative edges (representing positive and negative judgements). The edge signs were ignored in our analyses.

Advogato trust network **(`advogato`)**
A network of trust relationships among users on Advogato, an online community of open source software developers[11]. Edge directions indicate that node i trusts node j, and edge weight denotes one of four increasing levels of declared trust from i to j: observer (0.4), apprentice (0.6), journeyer (0.8), and master (1.0).

Epinions trust network **(`epinions_trust`)**
A who-trusts-whom social network of the general consumer review site Epinions.com[12]. Members can decide whether to "trust" each other. These trust relationships are combined with review ratings to determine which reviews are shown to the user.

PGP web of trust **(`pgp_strong`)**
Strongly connected component of the Pretty-Good-Privacy (PGP) web of trust among users, circa November 2009[2].

Physician trust network **(`physician_trust`)**
A network of trust relationships among physicians in four midwestern (USA) cities in 1966[13]. Edge directions indicate that node i trusts or asks for advice from node j. Each of the four components represent the network within a given city. We analyzed four (disconnected) components corresponding to different cities as separate networks.

E. coli transcription network **(`ecoli_transcription`)**
Directed network of operons and their pairwise interactions, via transcription factor-based regulation, within the bacteria Escherichia coli[14]. In our analyses we used v1.1 version and did not distinguish between different regulation types.

Yeast transcription network **(`yeast_transcription`)**
Directed network of operons and their pairwise interactions, via transcription factor-based regulation, within the yeast Saccharomyces cerevisiae[15]. We did not distinguish between different regulation types.

Collins yeast interactome **(`collins_yeast`)**
Undirected and unweighted network of protein-protein interactions in Saccharomyces cerevisiae (budding yeast), measured by co-complex associations identified by high-throughput affinity purification and mass spectrometry (AP/MS)[16].

Coulomb yeast interactome **(`interactome_yeast`)**
An undirected and unweighted network of protein-protein binding interactions among yeast proteins[17]. Nodes represent proteins found in yeast (Saccharomyces cerevisiae) and an edge represents a binding interaction between two proteins.

Figeys human interactome **(`interactome_figeys`)**
A directed unweighted network of human proteins and their binding interactions[18]. Nodes represent proteins and an edge represents an interaction between two proteins, as inferred using a mass spectrometry-based approach.

PDZ-domain interactome **(`interactome_pdz`)**
An undirected and unweighted network of PDZ-domain-mediated protein-protein binding interactions, extracted from the PDZBase database[19]. Nodes represent proteins and an edge represents a binding interaction between two proteins.

Joshi-Tope human protein interactome **(`reactome`)**
An undirected and unweighted network of human proteins and their binding interactions, extracted from Reactome project[20]. Nodes represent proteins and an edge represents a binding interaction between two proteins.

Stelzl human interactome **(`interactome_stelzl`)**
A directed unweighted network of human proteins and their binding interactions[21]. Nodes represent proteins and an edge represents an interaction between two proteins, as inferred via high-throughput Y2H experiments using bait and prey methodology.

Vidal human interactome **(`interactome_vidal`)**
An undirected and unweighted network of human proteins and their binding interactions[22]. Nodes represent proteins and an edge represents a binding interaction between two proteins, as tested using a high-throughput yeast two-hybrid (Y2H) system.

Table S4. Descriptive statistics ($N = 28$)

domain	dataset	network	s	c	n	S	ρ	$\langle d_i \rangle$	σ_{d_i}	$d_{\max}$
biological	collins_yeast		0.62	0.01	1004	0.62	0.02	16.57	1.12	127
	ecoli_transcription	v1.1	0.02	0.05	328	0.78	0.01	2.78	1.86	72
	interactome_figeys		0.01	0.14	2217	0.99	0.00	5.79	2.95	314
	interactome_pdz		0.00	0.21	161	0.76	0.02	2.60	1.12	21
	interactome_stelzl		0.01	0.12	1615	0.95	0.00	3.85	1.85	95
	interactome_vidal		0.04	0.03	2783	0.89	0.00	4.32	1.63	129
	interactome_yeast		0.05	0.01	1458	0.78	0.00	2.67	1.29	56
	reactome		0.61	0.05	5973	0.94	0.01	48.81	1.39	855
	yeast_transcription		0.02	0.19	664	0.72	0.00	3.21	1.79	71
social	advogato		0.09	0.02	5042	0.77	0.00	15.56	2.07	803
	dutch_criticism		0.16	0.22	35	1.00	0.13	4.57	0.65	12
	ego_social	facebook_combined	0.52	0.02	4039	1.00	0.01	43.69	1.20	1045
	epinions_trust		0.07	0.02	75877	1.00	0.00	10.69	4.02	3044
	facebook_friends		0.51	0.02	329	0.91	0.04	11.88	0.92	63
	facebook_organizations	L1	0.26	0.04	5793	1.00	0.00	10.62	1.73	320
		L2	0.22	0.02	5524	1.00	0.01	34.11	0.93	417
		M1	0.26	0.03	1429	1.00	0.02	27.09	1.06	339
		M2	0.23	0.02	3862	1.00	0.01	45.22	0.65	328
		S1	0.29	0.03	320	1.00	0.05	14.81	0.96	113
		S2	0.33	0.03	165	1.00	0.05	8.80	0.95	63
	karate	78	0.26	0.06	34	1.00	0.14	4.59	0.83	17
	pgp_strong		0.25	0.01	39796	1.00	0.00	9.91	2.46	1696
	physician_trust	1	0.17	0.05	117	1.00	0.07	7.95	0.50	26
		2	0.28	0.06	48	1.00	0.16	7.46	0.62	28
		3	0.32	0.08	41	1.00	0.17	6.93	0.42	15
		4	0.42	0.07	35	1.00	0.23	7.83	0.48	15
	residence_hall		0.30	0.04	217	1.00	0.08	16.95	0.46	56
	windsurfers		0.56	0.03	43	1.00	0.37	15.63	0.42	31

s - global similarity (clustering)
c - global complementarity
n - number of nodes in the giant component
S - relative size of the giant component
ρ - edge density
$\langle d_i \rangle$ - average node degree
σ_{d_i} - coefficient of variation of node degrees
$d_{\max}$ - maximum node degree

Networks used in "Similarity and complementarity in social relations"

Ugandan village networks `(ugandan_village)`

The dataset consists of unweighted and undirected networks of friendship and health advice relations between households in 17 rural villages bordering Lake Victoria in Mayuge District, Uganda. It has been originally studied in Ref.[23]. Relations were measured using the name generator approach in which a representative of each household was asked to indicate up to 10 persons considered friends or trustworthy in regard to health issues. Resulting ties were symmetrized.

Table S5. Descriptive statistics ($N = 34$)

domain	dataset	network	s	c	n	S	ρ	$\langle d_i \rangle$	σ_{d_i}	$d_{\max}$
social	ugandan_village	friendship-1	0.06	0.03	202	1.00	0.03	5.42	0.72	32
		friendship-2	0.11	0.05	181	0.99	0.04	7.60	0.77	44
		friendship-3	0.13	0.06	192	1.00	0.06	11.04	0.74	53
		friendship-4	0.09	0.05	320	1.00	0.04	12.97	0.66	50
		friendship-5	0.12	0.05	184	1.00	0.04	7.83	0.69	30
		friendship-6	0.14	0.07	139	1.00	0.07	9.09	0.68	42
		friendship-7	0.17	0.09	121	1.00	0.11	12.73	0.52	32
		friendship-8	0.06	0.04	369	1.00	0.03	9.50	0.70	58
		friendship-9	0.16	0.06	178	1.00	0.07	12.02	0.78	80
		friendship-10	0.10	0.07	207	1.00	0.05	10.55	0.64	44
		friendship-11	0.09	0.04	250	1.00	0.03	8.50	0.70	44
		friendship-12	0.08	0.05	229	1.00	0.03	7.62	0.86	58
		friendship-13	0.10	0.06	183	1.00	0.05	8.84	0.68	34
		friendship-14	0.15	0.06	124	1.00	0.07	8.47	0.64	36
		friendship-15	0.07	0.04	120	1.00	0.04	4.57	0.70	17
		friendship-16	0.05	0.03	372	1.00	0.02	7.38	0.72	43
		friendship-17	0.25	0.07	65	1.00	0.12	7.91	0.70	31
		health-advice_1	0.05	0.04	187	0.98	0.02	4.61	1.31	60
		health-advice_2	0.06	0.06	170	1.00	0.03	4.64	1.66	70
		health-advice_3	0.08	0.05	185	1.00	0.04	6.90	1.43	78
		health-advice_4	0.06	0.04	316	1.00	0.02	6.61	0.96	72
		health-advice_5	0.09	0.02	166	0.99	0.03	4.65	1.45	70
		health-advice_6	0.06	0.02	131	0.98	0.03	4.34	1.93	75
		health-advice_7	0.14	0.06	121	1.00	0.07	7.82	0.80	46
		health-advice_8	0.05	0.03	361	1.00	0.02	6.85	1.04	84
		health-advice_9	0.10	0.05	173	1.00	0.04	7.20	1.42	98
		health-advice_10	0.13	0.05	204	1.00	0.04	8.04	0.98	71
		health-advice_11	0.05	0.04	234	0.97	0.02	4.50	1.67	85
		health-advice_12	0.04	0.04	218	0.99	0.02	4.90	1.30	79
		health-advice_13	0.06	0.03	157	0.91	0.02	3.64	1.25	51
		health-advice_14	0.11	0.05	120	1.00	0.05	5.43	0.80	26
		health-advice_15	0.06	0.05	117	1.00	0.03	3.35	1.36	34
		health-advice_16	0.04	0.01	349	1.00	0.01	4.94	2.21	152
		health-advice_17	0.13	0.06	63	1.00	0.07	4.63	0.95	28
		**Average**	**0.09**	**0.05**	**197.29**	**0.99**	**0.04**	**7.21**	**1.01**	**56.09**

s - global similarity (clustering)
c - global complementarity
n - number of nodes in the giant component
S - relative size of the giant component
ρ - edge density
$\langle d_i \rangle$ - average node degree
σ_{d_i} - coefficient of variation of node degrees
$d_{\max}$ - maximum node degree

Networks used in "Structural diversity across the tree of life"

For this analysis we used a dataset of 1840 interactomes of different species across the tree of life published originally in Ref.[24]. The interactomes represent only physical protein-protein interactions that are experimentally supported or manually curated. Detailed description of the dataset and its underlying methodology, including the list of types of interactions that were considered, can be found in the Supplementary Information appendix of Ref.[24]. In particular, information on phylogenetic taxonomy information, the source of publication counts per species and evolution time estimates defined in terms of the number of nucleotide substitutions per site are discussed in sections S1.2, S2.2 and S3.

Due to the large number of networks we do not present a table with descriptive statistics here. The data and code used for conducting the analysis is available from the Github repository listed in the Main Text (Data and materials availability).

Experimental assessment of the performance of `PathCensus` algorithm

We assessed the performance of our implementation of `PathCensus` algorithm by measuring the average runtime for each of the 1840 interactome networks studied in the paper as well as their randomized counterparts sampled from UBCM. For each network an average runtime over 5 runs was calculated for the observed network and a corresponding randomized version sampled from UBCM (see Fig. S4).

Our analysis shows that the runtime scales approximately linearly with respect to $|E|\Delta S d_{\max}$, which agrees with the previous theoretical analysis of the computational complexity of `PathCensus` algorithm. However, the proportionality constant is lower for smaller networks and then increases for networks with about 100 nodes. After that, the linear scaling seems to be stable.

The experiment was run on a machine with Ubuntu (20.04.4 LTS) and Intel(R) Core(TM) i5-8300H CPU @ 2.30GHz. We did not use the parallelized version of `PathCensus` algorithm.

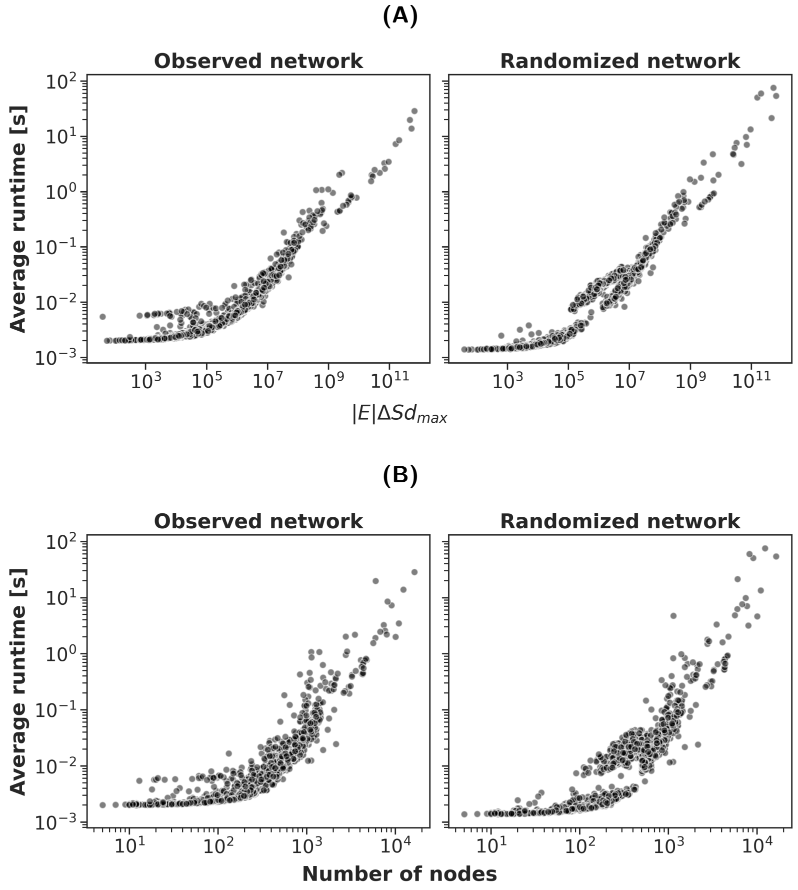

Figure S4. Experimental assessment of the performance of `PathCensus` algorithm. Computations used the $d_i \leq d_j$ optimization discussed in Algorithm S1. **(A)** Average runtime plotted against the product of the number of edges ($|E|$), maximum node degree ($d_{\max}$) and the maximum size of a Star$_i$ set (ΔS; see Algorithm S1). **(B)** Average runtime and network size.

References

1. Ahmed, N. K., Neville, J., Rossi, R. A. & Duffield, N. Efficient Graphlet Counting for Large Networks. In *2015 IEEE International Conference on Data Mining*, 1–10, DOI: 10.1109/ICDM.2015.141 (IEEE, Atlantic City, NJ, USA, 2015).

2. Richters, O. & Peixoto, T. P. Trust Transitivity in Social Networks. *PLoS ONE* **6**, e18384, DOI: 10.1371/journal.pone.0018384 (2011).

3. Peixoto, T. P. The Netzschleuder network catalogue and repository. https://networks.skewed.de/ (2020).

4. Fire, M. & Puzis, R. Organization Mining Using Online Social Networks. *Networks Spatial Econ.* **16**, 545–578, DOI: 10.1007/s11067-015-9288-4 (2016).
5. Maier, B. F. & Brockmann, D. Cover time for random walks on arbitrary complex networks. *Phys. Rev. E* **96**, 042307, DOI: 10.1103/PhysRevE.96.042307 (2017).
6. McAuley, J. & Leskovec, J. Learning to Discover Social Circles in Ego Networks. In *Advances in Neural Information Processing Systems*, vol. 25 (Curran Associates, Inc., 2012).
7. Zachary, W. W. An Information Flow Model for Conflict and Fission in Small Groups. *J. Anthropol. Res.* **33**, 452–473, DOI: 10.1086/jar.33.4.3629752 (1977).
8. Freeman, L. C., Webster, C. M. & Kirke, D. M. Exploring social structure using dynamic three-dimensional color images. *Soc. Networks* **20**, 109–118, DOI: 10.1016/S0378-8733(97)00016-6 (1998).
9. Freeman, L., Freeman, S. & Michaelson, A. On human social intelligence. *J. Soc. Biol. Syst.* **11**, 415–425, DOI: 10.1016/0140-1750(88)90080-2 (1988).
10. de Nooy, W. A literary playground: Literary criticism and balance theory. *Poetics* **26**, 385–404, DOI: 10.1016/S0304-422X(99)00009-1 (1999).
11. Massa, P., Salvetti, M. & Tomasoni, D. Bowling alone and trust decline in social network sites. In *2009 Eighth IEEE International Conference on Dependable, Autonomic and Secure Computing* (IEEE, Chengdu, China, 2009).
12. Richardson, M., Agrawal, R. & Domingos, P. Trust Management for the Semantic Web. In Fensel, D., Sycara, K. & Mylopoulos, J. (eds.) *The Semantic Web - ISWC 2003*, Lecture Notes in Computer Science, 351–368, DOI: 10.1007/978-3-540-39718-2_23 (Springer, Berlin, Heidelberg, 2003).
13. Coleman, J., Katz, E. & Mentzel, H. The Diffusion of an Innovation Among Physicians. *Sociometry* **20**, 253–270 (1957).
14. Shen-Orr, S. S., Milo, R., Mangan, S. & Alon, U. Network motifs in the transcriptional regulation network of Escherichia coli. *Nat. Genet.* **31**, 64–68, DOI: 10.1038/ng881 (2002).
15. Milo, R. *et al.* Network Motifs: Simple Building Blocks of Complex Networks. *Science* **298**, 824–827, DOI: 10.1126/science.298.5594.824 (2002).
16. Collins, S. R. *et al.* Toward a Comprehensive Atlas of the Physical Interactome of Saccharomyces cerevisiae. *Mol. & Cell. Proteomics* **6**, 439–450, DOI: 10.1074/mcp.M600381-MCP200 (2007).
17. Coulomb, S., Bauer, M., Bernard, D. & Marsolier-Kergoat, M.-C. Gene essentiality and the topology of protein interaction networks. *Proc. Royal Soc. B: Biol. Sci.* **272**, 1721–1725, DOI: 10.1098/rspb.2005.3128 (2005).
18. Ewing, R. M. *et al.* Large-scale mapping of human protein–protein interactions by mass spectrometry. *Mol. Syst. Biol.* **3**, 89, DOI: 10.1038/msb4100134 (2007).
19. Beuming, T., Skrabanek, L., Niv, M. Y., Mukherjee, P. & Weinstein, H. PDZBase: A protein-protein interaction database for PDZ-domains. *Bioinformatics* **21**, 827–828, DOI: 10.1093/bioinformatics/bti098 (2005).
20. Joshi-Tope, G. Reactome: A knowledgebase of biological pathways. *Nucleic Acids Res.* **33**, D428–D432, DOI: 10.1093/nar/gki072 (2004).
21. Stelzl, U. *et al.* A Human Protein-Protein Interaction Network: A Resource for Annotating the Proteome. *Cell* **122**, 957–968, DOI: 10.1016/j.cell.2005.08.029 (2005).
22. Rual, J.-F. *et al.* Towards a proteome-scale map of the human protein–protein interaction network. *Nature* **437**, 1173–1178, DOI: 10.1038/nature04209 (2005).
23. Chami, G. F., Ahnert, S. E., Kabatereine, N. B. & Tukahebwa, E. M. Social network fragmentation and community health. *Proc. Natl. Acad. Sci.* **114**, E7425–E7431, DOI: 10.1073/pnas.1700166114 (2017).
24. Zitnik, M., Sosič, R., Feldman, M. W. & Leskovec, J. Evolution of resilience in protein interactomes across the tree of life. *Proc. Natl. Acad. Sci.* **116**, 4426–4433, DOI: 10.1073/pnas.1818013116 (2019).
